# Migration through a Major Andean Ecogeographic Disruption as a Driver of Genetic and Phenotypic Diversity in a Wild Tomato Species

**DOI:** 10.1093/molbev/msab092

**Published:** 2021-04-03

**Authors:** Jacob B Landis, Christopher M Miller, Amanda K Broz, Alexandra A Bennett, Noelia Carrasquilla-Garcia, Douglas R Cook, Robert L Last, Patricia A Bedinger, Gaurav D Moghe

**Affiliations:** 1 Department of Botany and Plant Sciences, University of California, Riverside, Riverside, CA, USA; 2 Plant Biology Section, School of Integrative Plant Science, Cornell University, Ithaca, NY, USA; 3 Department of Biology, Colorado State University, Fort Collins, CO, USA; 4 Department of Plant Pathology, University of California, Davis, Davis, CA, USA; 5 Department of Biochemistry and Molecular Biology, Michigan State University, East Lansing, MI, USA; 6 Department of Plant Biology, Michigan State University, East Lansing, MI, USA

**Keywords:** RAD-seq, population genomics, mating systems, plant evolution, metabolic evolution, biodiversity

## Abstract

Evolutionary dynamics at the population level play a central role in creating the diversity of life on our planet. In this study, we sought to understand the origins of such population-level variation in mating systems and defensive acylsugar chemistry in *Solanum habrochaites*—a wild tomato species found in diverse Andean habitats in Ecuador and Peru. Using Restriction-site-Associated-DNA-Sequencing (RAD-seq) of 50 *S. habrochaites* accessions, we identified eight population clusters generated via isolation and hybridization dynamics of 4–6 ancestral populations. Detailed characterization of mating systems of these clusters revealed emergence of multiple self-compatible (SC) groups from progenitor self-incompatible populations in the northern part of the species range. Emergence of these SC groups was also associated with fixation of deleterious alleles inactivating acylsugar acetylation. The Amotape-Huancabamba Zone—a geographical landmark in the Andes with high endemism and isolated microhabitats—was identified as a major driver of differentiation in the northern species range, whereas large geographical distances contributed to population structure and evolution of a novel SC group in the central and southern parts of the range, where the species was also inferred to have originated. Findings presented here highlight the role of the diverse ecogeography of Peru and Ecuador in generating population differentiation, and enhance our understanding of the microevolutionary processes that create biological diversity.

## Introduction

How new phenotypes emerge and evolve in populations has been a long-standing question in evolutionary biology (Darwin 1859; Mayr 1942; Schlichting 1986; Reznick and Ricklefs 2009; Harvey et al. 2019). Over the past decade, high-throughput sequencing has enabled integration of phylogenetic and mechanistic studies of phenotypic variation across populations, improving our understanding of microevolution (Andrews et al. 2016; Harvey et al. 2019). In this study, we investigated the evolution of significant reproductive and metabolic variation observed in the wild tomato species *Solanum habrochaites*, found in Peruvian and Ecuadorian Andes. Using Restriction site Associated DNA Sequencing (RAD-seq) (Miller et al. 2007; Baird et al. 2008) in conjunction with phenotyping of mating systems and defensive metabolites called acylsugars, we sought to characterize how this phenotypic variation was shaped by the evolutionary history of the species and the unique geography of the Andes.

*Solanum habrochaites* (Knapp and Spooner 1999) is a phenotypically diverse species with a range from the upper reaches of the Atacama desert in southern Peru to the tropical forests of central Ecuador. Tracking the western slope of the Andean mountain range in the south, this species is generally found 1,000–3,000 m above sea level but also extends to sea level in central Ecuador. This species range overlaps with the tropical Andes biodiversity hotspot, home to a sixth of global plant life and >20,000 endemic plant species (Myers et al. 2000; Hazzi et al. 2018). Understanding the origins of genetic and phenotypic diversity in *S. habrochaites* can thus provide molecular mechanistic insights into the origins of biological diversity in this region.

Previous studies in this species (Gonzales-Vigil et al. 2012; Kim et al. 2012; Schilmiller et al. 2015; Fan et al. 2017) have demonstrated substantial variation in two trichome-localized compound classes—acylsugars and terpenes—that are important for defense against herbivores (Weinhold and Baldwin 2011; Leckie et al. 2016). For example, *S. habrochaites* accessions were grouped into two chemotypic superclusters based on their acylsugar profiles—a “northern” supercluster that failed to add an acetyl (C2) group to the sucrose R2 position in acylsugars, and a “southern” supercluster that retained this activity (Kim et al. 2012). This loss of C2 addition resulted from mutational inactivation of acylsugar acyltransferase 4 (ASAT4)—the final enzyme in the *Solanum* acylsugar biosynthetic pathway—occurring via different means. Another study demonstrated differential acylation between northern and southern accessions on the furanose ring of the acylsugar, which could be traced back to gene duplication, divergence and loss in ASAT3, an upstream enzyme in the pathway (Schilmiller et al. 2015). However, demographic processes that influenced this evolution of acylsugar profiles remain unknown.

*Solanum habrochaites* is also an attractive system for the study of reproductive trait evolution, with extensive diversity both in mating system and in reproductive barriers that affect interpopulation and interspecific gene flow. *Solanum habrochaites* is predominantly an obligate outcrossing species due to gametophytic S-RNase-based self-incompatibility (SI) (Mutschler and Liedl 1994; Peralta et al. 2008; Bedinger et al. 2011). In this type of SI, the *S*-locus encodes pistil-expressed S-RNases and pollen-expressed *S*-locus F-box proteins that determine the specificity of the SI interaction. In addition, other pistil-expressed (e.g., HT protein) and pollen-expressed (e.g., CUL1) factors that are not linked to the *S*-locus play a role in self pollen rejection (reviewed in Bedinger et al. 2017). SI is widespread in flowering plants, and acts to preserve genetic diversity and diminish inbreeding depression (Stebbins 1957; Lande and Schemske 1985; Schemske and Lande 1985; Takayama and Isogai 2005; Igic et al. 2008). However, there may be a selective advantage for transitions to self-compatibility (SC) during the dispersal of species, since a single SC individual could conceivably colonize a novel environment in the absence of other individuals or pollinators (Baker 1955; Stebbins 1957; Baker 1967; Pannell et al. 2015).

SC populations of *S. habrochaites* have been identified at the northern and southern species range margins (Martin 1961; Rick et al. 1979) in Ecuador and southern Peru, respectively. These marginal SC populations represent independent SI to SC transitions (Rick and Chetelat 1991). Groups of SC populations at the northern species margin exhibit diverse reproductive barriers acting at the individual, population, and species levels (Martin 1961; Broz, Randle, et al. 2017). Previous work that combined reproductive trait data with sequence analysis of *S-RNase* alleles identified two distinct SC groups (SC-1 and SC-2), and suggests that SC has arisen at least twice at the northern margin (Broz, Randle, et al. 2017).

The ancestral SI populations of *S. habrochaites* originated in northern/central Peru (Rick et al. 1979; Peralta et al. 2008; Pease et al. 2016) and, as the species spread, it traversed the Amotape-Huancabamba Zone (AHZ)—a region of cordillera disruption bordered in the south by Río Chicama in Peru and in the north by Río Jubones in Ecuador. This geographical disruption—one of the lowest altitude regions in tropical Andes—was generated through repeated geological fragmentation and remodeling over millions of years (Antonelli et al. 2009; Hoorn et al. 2010), creating a number of unique microhabitats and rare east-west passages for species movement in the Andes. The AHZ includes a floristically diverse region called the Huancabamba Depression (HD) in the central part of the AHZ, which contains the lowest point in the entire Peruvian Andes (Weigend 2002). The HD has sometimes been referred to (Richter et al. 2009)—controversially (Weigend 2004; Mutke et al. 2014)—as a barrier to dispersal of some high altitudinal plant species due to its low-lying nature. The species diversity in the overall AHZ is 6-8 times higher than in the area adjacent to the AHZ (Weigend 2002). With its highly variable microhabitats and low-lying geology, the AHZ also shows high rates of endemism for both plants and animals (Berry 1982; Weigend 2002), and may have also influenced *S. habrochaites* evolution.

To determine how both SI to SC transitions and acylsugar diversification occurred in the context of *S. habrochaites* species range expansion, we first determined the species’ population structure using RAD-seq and studied patterns of gene flow between different populations. We identified additional independent SI to SC transitions at the northern species margin, which were also associated with evolution of new acylsugar phenotypes. Our results revealed that alleles that eventually led to fixation of these novel phenotypes in Ecuador first emerged in the AHZ during the northward migration of *S. habrochaites* from central Peru. In contrast, we found a greater impact of geographical distance in central/southern Peru in the production of locally isolated populations. This work underscores the critical role of ecogeography and of repeated evolution of SC in shaping biological diversity.

## Results

### Preliminary Analysis of RAD-Seq Data

For RAD-seq, we selected 52 out of the >100 accessions of *S. habrochaites* in the TGRC germplasm database (https://tgrc.ucdavis.edu/, last accessed April 01, 2021) that span the entire species range—a distance of ∼2,000 km from Jipijapa, Ecuador (accession LA1625) in the north to Ocaña District, Peru in the south (accession LA1928). Four *Solanum pennellii* accessions were included as outgroups. As expected, there was large variation in the number of reads obtained per sample (supplementary fig. S1*A*, Supplementary Material online), ranging from 5,707 to 5,348,246. After filtering the initial reads based on quality, one or both replicates of five accessions—LA2868, LA2976, LA1978, LA2098, LA0716—were removed (supplementary fig. S1*A*, Supplementary Material online), leaving a total of 53 accessions for further analyses (supplementary file S1, Supplementary Material online). We did not find any correspondence between number of reads between technical replicates (supplementary fig. S1*B*, Supplementary Material online) suggesting that the read number variation was due to the randomness associated with restriction cleavage and the RAD-seq library preparation protocol as opposed to within-species variation of restriction sites. To improve read coverage per accession, we combined reads from the technical replicates and mapped all reads to the draft-quality *S. habrochaites* LYC4 genome (Aflitos et al. 2014) to make clustered read stacks (Catchen et al. 2013). The number of retained loci and mean coverage across all loci also varied across accessions (supplementary fig. S1*C* and *D*, Supplementary Material online). The median read coverage across all sites after filtering was 9.5, with 20 out of 53 accessions having a mean read coverage ≥10× (supplementary fig. S1*D*, Supplementary Material online).

### Eight Population Clusters Can Be Identified in the Sampled *S. habrochaites* Accessions

**Fig. 1.**
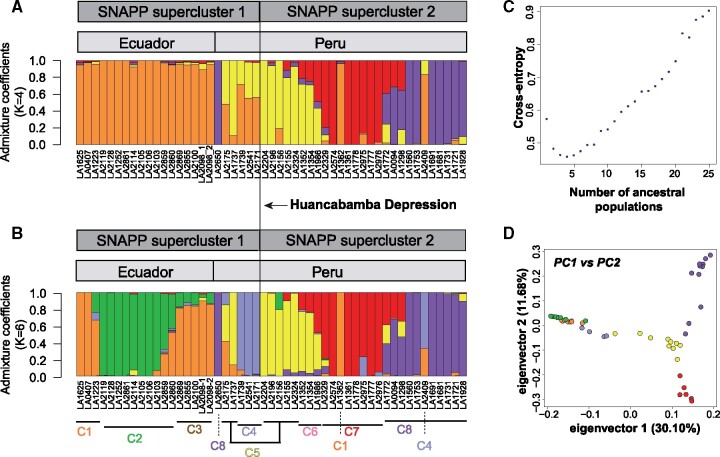
Population structure of *Solanum habrochaites*. (*A*, *C*) Population structure plots obtained using *K* = 4 and *K* = 6 as predefined number of genetic clusters using Set 1 SNPs. Population cluster numbers as per figure 2*A* are noted below the bar plot in (*C*). (*B*) Cross-entropy criterion showing *K* = 4 as the optimal number of genetically differentiated ancestral populations. (*D*) Principal components analysis, with populations defined based on *K* = 6. Color corresponds to the major ancestral population predicted in subfigure *C*.

To assess the genetic relatedness between *S. habrochaites* accessions across the range, we first inferred SNP-genotype based ancestral populations (*K*) using three different SNP data sets. These results suggested that the sampled *S. habrochaites* individuals arose from 4 to 6 ancestral populations (fig. 1; supplementary fig. S2, Supplementary Material online)—three of which (*purple, red, yello*w) lie south of the AHZ. While *K* = 4 was the optimal cluster size based on the cross-entropy criterion (fig. 1*B*), manual observation of population assignments at multiple *K*s (supplementary fig. S2, Supplementary Material online), previous results using simple sequence repeat (SSR) markers (Sifres et al. 2011) and empirical observations of *S. habrochaites* reproductive and metabolic diversity led us to also consider the possibility of six ancestral populations (fig. 1*C*). In Ecuador, the optimal *K* = 4 suggested presence of one ancestral *orange* population (fig. 1) while *K* = 6 discriminated individuals in this region as originating from two different ancestral populations—*orange* and *green—*with different degrees of genotype sharing between them (fig. 1*C*). We also observed that multiple accessions—at both *K* = 4 and *K* = 6 values—across the range had small yet non-negligible probabilities of being assigned to *yellow*, *red*, and *purple* populations, fixed south of the HD.

**Fig. 2.**
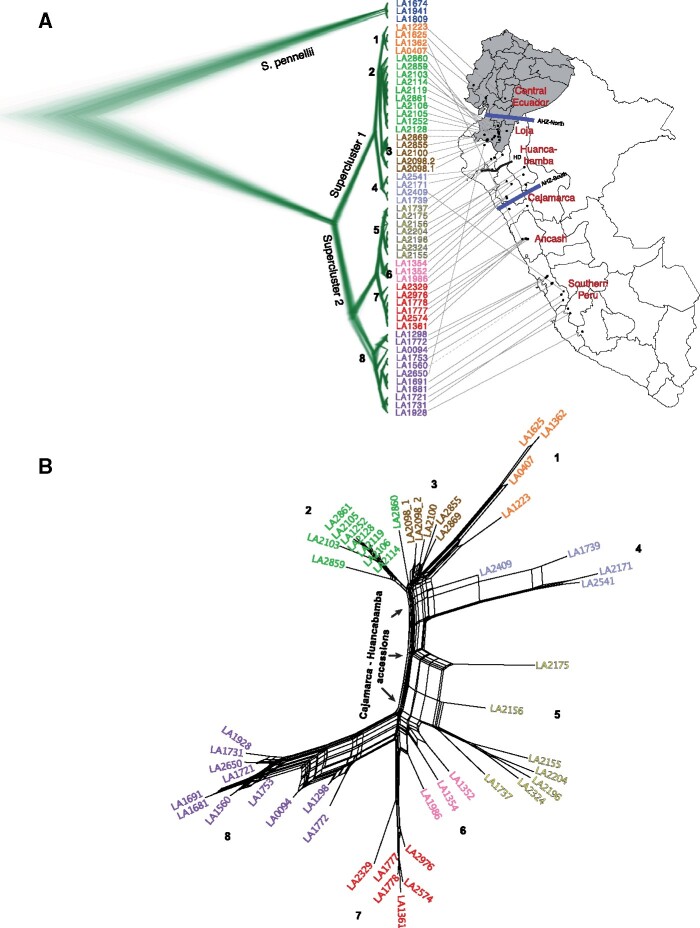
Coalescent and migration analysis. (*A*) Results of coalescent analysis using SNAPP, obtained using markers shared between all sampled individuals. LA2975 was left out from this analysis because its level of heterozygosity was >3× the next highest sample, suggesting possible contamination or other unexplained behavior. AHZ, Amotape-Huancabamba Zone; HD, Huancabamba Depression. Population cluster numbers are marked within the phylogeny. Region names in red as per Sifres et al. (2011) and do not represent provinces. (*B*) A split network of the population clusters shows close relationships within the northern and within southern clusters and significant variation in the Cajamarca and Huancabamba accessions. Significant incongruencies are seen for clusters 4–6 and three accessions of cluster 8. AHZ, Amotape-Huancabamba Zone.

PCA using Set 1 and 2 markers (fig. 1*D*;supplementary fig. S3, Supplementary Material online) showed a close relationship between accessions north of the HD, but interestingly in the south, the southernmost *purple* individuals were more related to *yellow* individuals near the central part of the range—near Huancabamba/Cajamarca—than to their geographically close *red* individuals. To better understand this observation, we employed coalescent tree (Bryant et al. 2012) and network-based approaches. The coalescence-based approach, obtained using 3,965 markers represented in all *S. habrochaites* and *S. pennellii* accessions, infers Bayesian trees for every SNP identified in the population and integrates for coalescence across all trees. Eight genotypically distinct population clusters within *S. habrochaites* (fig. 2*A*) were identified, largely following the clusters inferred from structure analysis at *K* = 6 (fig. 1*C*). The two additional clusters 3 and 6 comprised hybrids between the *green-orange* and *red*-*yellow* populations (fig. 1*C*). Population clusters 1–4 (supercluster 1; northern supercluster) correspond to samples from mid/southern Ecuador and northern Peru, while clusters 5–8 (supercluster 2; southern supercluster) correspond to samples in central and southern Peru (fig. 2*A*). The two superclusters are separated at the HD. The coalescent tree also supported the PCA finding that cluster 8 was not closely related to its geographical neighbor cluster 7 but was genetically equidistant from clusters 5 to 7.

Split network-based approaches are used to model events, such as gene loss/duplication, horizontal gene transfer, recombination, hybridization as well as systematic errors that are typically not modeled in a phylogenetic tree. The systematic errors in model-based tree reconstructions—multiple parallel edges in split networks are indicative of incompatibilities between tree-based phylogenetic relationships (Huson and Bryant 2006). A split network of *S. habrochaites* accessions revealed that clusters 4–6 in the Huancabamba/Cajamarca region and three accessions from cluster 8 (LA1772, LA0094, LA1298) exhibit substantial incompatibilities (fig. 2*B*). In the coalescent tree-based analysis, the three cluster 8 accessions formed a distinct subgroup (fig. 2*A*), and the structure analysis suggested that all contain a mixture of three genotypes (*purple, red, yellow*; fig. 1*C*). These cluster 8 relationships are further clarified below when the mating systems of the different accessions are taken into account. Nonetheless, based on the above results and considering previous knowledge that *S. habrochaites* originated further north near the AHZ (Rick et al. 1979; Sifres et al. 2011; Pease et al. 2016), we propose that cluster 8 was established largely by southward migration from near the AHZ (clusters 5, 6). Under this scenario, cluster 7 would result from an independent, local fixation of the *red* genotype, and would explain results from all four analyses above. This scenario also allows for a rapid separation of the two superclusters via northward/southward migrations, as seen in the coalescent analysis (fig. 2*A*) and previous studies (Rick et al. 1979; Sifres et al. 2011; Pease et al. 2016).

Taken together, the four methods suggest that there are eight current population clusters across the sampled *S. habrochaites* accessions, likely derived from 4 to 6 ancestral populations. Three clusters 1, 7, 8 were well-differentiated in all analyses performed, clusters 2–3 and 5–6 were found to be more similar to each other, and cluster 4—in the heart of the AHZ—is genetically substantially differentiated from other clusters. We note that six of the eight population clusters occur north of the southern boundary of the AHZ, while only clusters 7, 8 occur entirely south of the AHZ. This finding is consistent with the notion of AHZ as a diversifying force. Furthermore, several of the observed population clusters reflect the distribution of distinct SC populations identified in previous studies (Rick et al. 1979; Broz, Randle, et al. 2017), offering a possible mechanistic explanation for the existence of these clusters. We thus examined reproductive traits to evaluate the contribution of mating systems to population structure.

### Reproductive Traits in *S. habrochaites* as Drivers of Population Differentiation

Reproductive traits including mating system can directly impact reproductive isolation of populations. Mating system transitions from SI to SC are a common evolutionary event in plants (Stebbins 1974), especially at species margins where dispersal into a new environment can create a population bottleneck. In this scenario, mates can become limiting, resulting in strong selection for SC (Baker 1955, 1967) with subsequent reduction in gene flow between newly established SC populations and their ancestral SI populations.

*Solanum habrochaites* displays substantial diversity in the expression of reproductive traits (Martin 1961, 1963; Rick et al. 1979; Mutschler and Liedl 1994; Sacks and St. Clair 1998; Covey et al. 2010; Baek et al. 2015; Markova et al. 2016; Broz, Randle, et al. 2017) (https://tgrc.ucdavis.edu/, last accessed April 01, 2021). The SC-1 and SC-2 groups previously reported at the northern *S. habrochaites* species margin (Broz, Randle, et al. 2017) aligned well with coalescent clusters 2 and 1, respectively. We therefore thoroughly analyzed reproductive traits in additional *S. habrochaites* accessions to establish trait groups and align these with population structure. Seven reproductive traits—mating system, S-RNase expression, *S-RNase* allele, HT protein expression, pollen- and pistil-side interpopulation barriers, and unilateral incompatibility (UI) between *S. habrochaites* and other tomato clade species—were analyzed, enabling us to produce a comprehensive inventory of reproductive traits for 34 accessions throughout the *S. habrochaites* range (table 1). We confirmed an SC mating system for 19 accessions at the northern species margin in Ecuador and for four accessions at the southern species margin. Further analysis of reproductive traits allowed identification of distinct SC groups (supplementary text 1, Supplementary Material online).

**Table 1.** Reproductive Traits for *Solanum habrochaites* Accessions.

Accession	Mating System and SC Group	S-RNase Protein	*S-RNase* Allele(s)	HT Protein	Interpop pollen-side	Interpop pistil-side	UI	RAD-Seq Cluster
LA4656	SC-2	N	*LhgSRN-1*	Y	Y	N	Y	na
LA1624^a^	SC-2	N	*LhgSRN-1*	Y	Y	N	Y	C1b
PI129157^a^	SC-2	N	*LhgSRN-1*	Y	Y	N	Y	C1^b^
LA1625^a^	SC-2	N	*LhgSRN-1*	Y	Y	N	Y	C1
LA1266^a^	SC-1	N	*hab-7*	Y	N	N	Y	na
PI134417	SC-2	N	*LhgSRN-1*	Y	nt	N	nt	C1^b^
LA1264^a^	SC-1	N	*hab-7*	Y	N	N	Y	na
PI390515	SC-2/3	N	*LhgSRN-1*	N	Y	N	N	C1^b^
LA0407^a^	SC-2	N	*LhgSRN-1*	Y	Y	N	Y	C1
LA1223^a^	SC-3	N	*LhgSRN-1*	N	N	N	N	C1
PI251305	SC-1/2/3	Y	*hab-7/LhgSRN-1*	N	N	Y	Y	C1^b^
LA4654	SC-6	N	Unknown	Y	N	N	Y	na
LA4655	SC-6	N	Unknown	low	N	N	Y	na
LA2119^a^	SC-1	N	*hab-7*	Y	N	N	Y	C2
LA2868^a^	SI	Y	Multiple^c^	Y	N	Y	Y	C1^b^
LA2128	SC-1	N	*hab-7*	Y	N	N	Y	C2
LA1252	SC-1	N	*hab-7*	Y	N	N	Y	C2
LA2855^d^	MP	Y	Multiple	Y	N	Y	Y	C3
LA2106^a^	SC-1	N	*hab-7*	Y	N	N	Y	C2
LA2101^a^	SC-5	N	Unknown	Y	N	N	Y	C3^b^
LA2860	SC-5	N	Unknown	Y	N	N	Y	C2
LA2864^a^	SI	Y	Multiple	Y	N	Y	Y	C3^b^
LA2099^a^	MP	Y	Multiple^c^	Y	N	Y	Y	C3^b^
LA2098^a^	MP	Y	Multiple	Y	N	Y	Y	C3
LA2175^a^	MP	Y	Multiple	Y	N	Y	Y	C5
LA1391^a^	MP	Y	Multiple^c^	Y	N	Y	Y	C4^b^
LA2314^a^	SI	Y	Multiple	Y	N	Y	Y	na
LA1353^a^^,^^e^	SI	Y	Multiple^c^	Y	N	Y	Y	na
LA1777^a^^,^^e^	SI	Y	Multiple	Y	N	Y	Y	C7
LA0094^d^	MP	Y	Multiple^c^ inc *hab-6*	Y	N	Y	Y	C8
LA1560^d^	SC-4	Y	*hab-6*	nt	N	nt	nt	C8
LA1753^d^	SC-4	Y	*hab-6*	Y	N	nt	nt	C8
LA1691	SC-4	Y	*hab-6*	nt	N	nt	Y	C8
LA1927^e^	SC-4	Y	*hab-6*	Y	Y	Y	Y	C8^b^

Note.—Reproductive traits documented for each accession include mating system as detected by fruit production after self-pollination and/or pollen tube growth analysis (SC, self-compatible; SI, self-incompatible); expression of S-RNase protein as detected by immunoblotting; *S-RNase* allele as detected by allele-specific PCR. Expression of HT protein as detected by immunoblotting; pollen-side interpopulation reproductive barriers as detected by pollen tube growth in crosses with pollen from different accessions onto pistils of SI accession LA1777 (Y = pollen tubes rejected, N = pollen tubes accepted); pistil-side interpopulation barriers as detected by pollen tube growth with pollen of SC accession LA0407 onto pistils of different accessions (Y = pollen rejected, N = pollen accepted); interspecific unilateral incompatibility (UI) detected by pollen tube growth in crosses using cultivar (*Solanum lycopersicum*) pollen onto pistils of different accessions (Y = pollen tubes rejected, N = pollen tubes accepted); and SC group based on the combination of reproductive traits and *S-RNase* allele present.

a Data from Broz, Randle, et al. (2017).

b By inference, due to either TSS data or due to geographic location, na = not applicable because no accessions from this region were included in the RAD-seq analysis, nt = not tested. Unshaded portion of the table shows accessions from Ecuador and shaded portion of the table shows accessions from Peru.

c Presence of *LhgSRN-1* allele detected at a low frequency.

d Mating system reported here differs from TGRC designation.

e Data from Covey et al. (2010).

The expression of functional S-RNase proteins is required for SI, and we found that all SI and mixed SI/SC population (MP) accessions expressed S-RNases, as expected. In contrast, S-RNase proteins were not detectable in styles of the northern SC accessions (with the exception of PI251305) in agreement with previous findings (Broz, Randle, et al. 2017). The southern marginal SC accessions expressed S-RNase protein, as has previously been shown for the southern SC accession LA1927 (Covey et al. 2010). In several cases, specific *S-RNase* alleles could be correlated with SC (table 1). In nine SC accessions in western coastal to central mountainous regions of Ecuador, the low-expression *LhgSRN-1* allele is associated with SC-2 and SC-3 groups (Kondo et al. 2002; Covey et al. 2010; Broz, Randle, et al. 2017). In a north-south corridor centered on Loja, the newly identified low-expression *hab-7 S-RNase* allele (supplementary fig. S5, Supplementary Material online) is associated with seven SC-1 group accessions. At the southern species margin, the expressed but low-activity *hab-6 S-RNase* allele (Covey et al. 2010) was detected in all four SC accessions tested, and this SC group was designated as SC-4.

Expression of HT protein, a pistil-specific SI and UI factor (McClure et al. 1999; Tovar-Méndez et al. 2014, 2017), was detected in styles of all tested accessions except for three from central Ecuador (LA1223, PI390515, and PI251305). PCR amplification and sequencing of the *HT-A* gene demonstrated that these three Ecuadorian accessions all have the same nonsense mutation in the second exon of the gene (supplementary fig. S6, Supplementary Material online).

Since interpopulation reproductive barriers based on pollen tube rejection in styles also act to reduce gene flow and allow for population differentiation, we tested for this reproductive trait. Unidirectional interpopulation barriers between central SI *S. habrochaites* populations and the northernmost (SC-2) SC populations have been documented (Martin 1963; Broz, Randle, et al. 2017) and are associated with the mutational loss of pollen-side SI factors (Markova et al. 2016). In this study, we found that pollen tubes of two previously uncharacterized northern accessions (LA4656 and PI309515) were rejected in styles of SI accessions, indicating the presence of pollen-side interpopulation barriers in these accessions, like those present in the SC-2 group. In addition, we confirmed that any accessions (either SI or SC) that express any S-RNase protein reject SC-2 group pollen tubes in styles and thus possess pistil-side interpopulation barriers (Broz, Randle, et al. 2017) (supplementary fig. S4, Supplementary Material online; table 1).

The final reproductive trait that we analyzed was the presence/absence of UI reproductive barriers between *S. habrochaites* and other tomato clade species. The absence of UI barriers would impact population structure if *S. habrochaites* hybridized with co-occurring tomato clade species, such as *Solanum pimpinellifolium*. A previous study identified a single *S. habrochaites* accession (LA1223, group SC-3) that lacked interspecific barriers (Broz, Randle, et al. 2017). In this study, we found one additional SC accession, PI390515, which lacked UI barriers (table 1; supplementary fig. S4, Supplementary Material online) but 29 other accessions tested had intact UI barriers.

Based on our phenotyping results, we were able to distinguish four SC groups, SC-1, SC-2, SC-3, and SC-4. Surprisingly, in one region of central Ecuador near Alausí, we found different combinations of reproductive traits including *S-RNase* alleles, HT expression, interpopulation barriers and interspecific barriers associated with the SC-1, SC-2, and SC-3 groups, suggesting that these SC groups meet and interbreed in this area. In addition, there were two other geographically distinct sets of SC accessions (one near Girón, Ecuador and one near Cariamanga, Ecuador) with unknown *S-RNase* alleles, whose relationships to other SC groups were unclear.

After delineating the reproductive trait groups, we evaluated the association of these groups with the population clusters. Because there were several accessions of significant phenotypic interest that were not included in the RAD-seq population genetic study, we utilized Targeted Sanger Sequencing (TSS) of 22 broadly represented and high coverage polymorphic RAD-seq loci to incorporate these accessions into the population study.

**Fig. 3.**
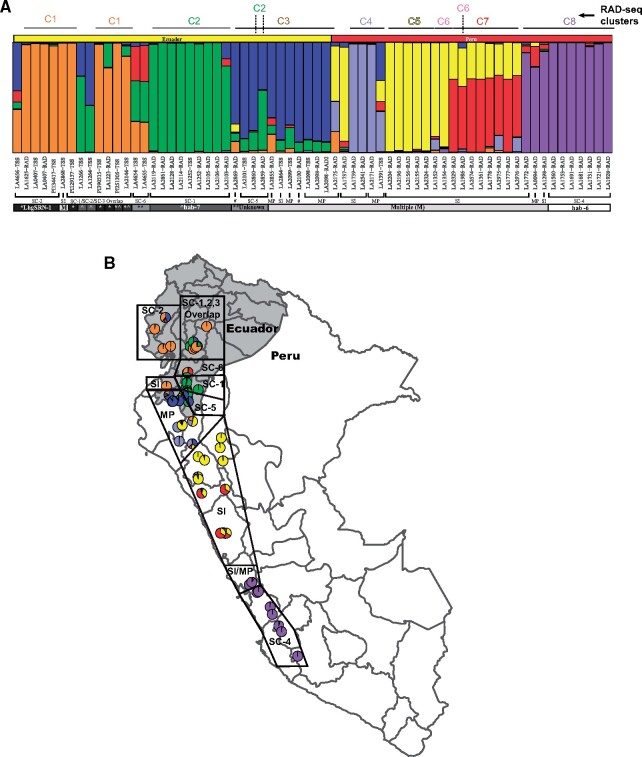
Mating system and population structure in *Solanum habrochaites*. (*A*) Population structure plot incorporating accessions analyzed using RAD-seq and TSS organized in a north to south array from left to right. Coalescent cluster IDs are noted at the top of the structure plot. Mating systems indicated include SC groups 1–6 (table 1), Mixed Population (MP) accessions containing both SI and SC individuals as well as purely SI accessions (SI). #, mating system not assessed. Where known, specific *S-RNase* alleles associated with accessions are indicated. *accessions containing the *LhgSRN-1 S-RNase* allele, ^accessions containing the *hab-7 S-RNase allele*, **S-RNase allele unknown, and multiple (M) *S-RNase* alleles are found in SI and MP accessions. (*B*) Map of Ecuador and Peru displaying the locations of accessions with different mating systems as shown in (*A*) and listed in table 1.

When the TSS and RAD-seq data for the 22 loci were combined and analyzed, seven ancestral populations could be identified that corresponded closely with reproductive traits (fig. 3*A*). The distinct coalescent cluster 1 (*orange*; figs. 2*A* and 3*A*) mapped to the SC-2 group and includes accessions found at the northernmost species margin along a steep altitudinal cline in central Ecuador (fig. 3*B*). Notably, the northernmost SI accession in Ecuador, LA2868, which contains the *LhgSRN-1 S-RNase* allele at a low frequency (table 1) also clusters with this group and may therefore represent an ancestral SI population. Another distinct cluster (*green*; figs. 2*A* and 3*A*) contained SC-1 group accessions in northeastern Loja (fig. 3*B*).

The relationship of SC-1/SC-2 to other SC groups was clarified by the population analysis. Two SC accessions collected west of the town of Gíron in central Ecuador LA4654 and LA4655 (fig. 3*B*), share a unique polymorphism pattern in the TSS analysis (fig. 3*A*, *red/green/orange*) and these accessions were tentatively designated as group SC-6 (table 1). The SC group near Cariamanga (*blue*, fig. 3*A*) clusters with SI and MP accessions but does not cluster with other SC groups. Therefore, SC accessions in this cluster were tentatively designated as group SC-5. In the full population structure analysis, this cluster appeared as a hybrid between *orange* and *green* genotypes (fig. 1*C*), but is resolved into an independent population with the 22 TSS markers. The co-clustering of SC, MP and SI accessions in the *blue* cluster suggests that the emergence of distinct SC-5 and MP groups in southwest Ecuador region (fig. 3*B*) is recent enough that only moderate genetic differentiation has occurred between these groups and their SI progenitors.

The population structure results using both RAD-seq and TSS markers are also consistent with the phenotyping results suggesting that different SC groups can interbreed when they encounter each other in the region noted as SC-1/SC-2/SC-3 overlap (fig. 3*B*). The finding of multiple Ecuadorian SC groups with distinct genetic and reproductive characters suggests that northward migration through the geographically disruptive AHZ, combined with recurring, independent, rounds of selection for SC under conditions of mate limitation, led to population differentiation at the northern species margin.

In southern Peru, the SC-4 group with the *hab-6 S-RNase* allele (Covey et al. 2010) clusters with two SI accessions (LA1772 and LA1298) and one MP accession (LA0094) from the same region (fig. 3*A*, *B*, *purple*). The emergence of the SC-4 group could have occurred from an MP type population (e.g., similar to LA0094) as a result of selection for reproductive assurance as the species migrated southward. Alternatively, LA0094 could be a hybrid between the *purple* SC and the SI sub-populations. Further experiments will be needed to address the origins of the MP individuals.

Overall, our detailed phenotyping of reproductive traits suggests that the six population clusters identified in the AHZ are a result of 1) population differentiation of SI clusters 3, 4 and 5, and 2) the independent establishment of multiple different SC groups. The unidirectional reproductive barriers seen between some populations further strengthened reproductive isolation, resulting in population differentiation between some SC and SI groups. Therefore, using the larger RAD-seq data set, we asked how the independent emergence of SC has influenced gene flow between different clusters in the context of the unique geographical features of Ecuador and Peru.

### Characterizing Population Differentiation in *S. habrochaites*

The characterization of mating systems across the *S. habrochaites* range allows us to ask two questions using genome-wide RAD-seq data: 1) what is the extent of gene flow between the different SC and SI/MP clusters?, and 2) which other evolutionary mechanisms contributed to *S. habrochaites* population structure besides emergence of SC? Three properties were assessed to address these questions—heterozygosity, population differentiation, and probability of isolation by distance (IBD). We also tested the effect of historical migration between clusters, but this was not found to be a major contributor to population structure (supplementary fig. S8*B* and supplementary text 1, Supplementary Material online).

**Fig. 4.**
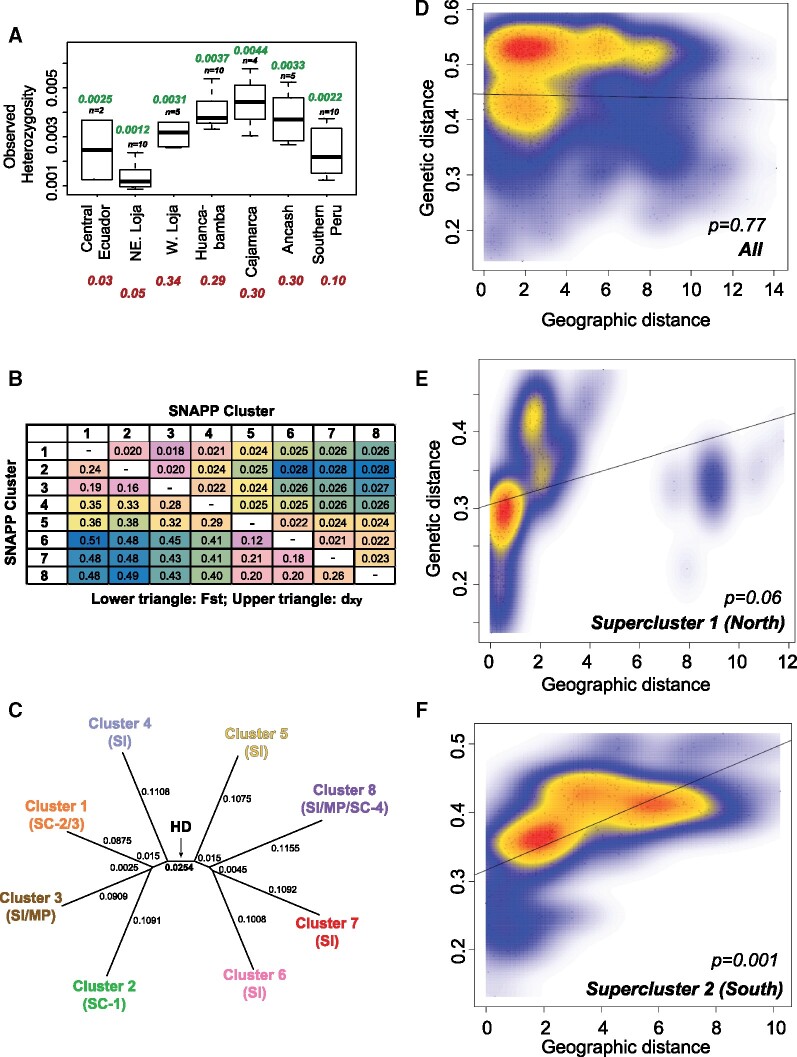
Analysis of population relatedness and demographic events (*A*) Observed heterozygosity estimates based on all genome-wide RAD mapping sites, for individuals classified by their geographic regions. Northeastern Loja and Western Loja all comprise individuals assigned to clusters 2 and 3, respectively. Number in green above the boxplot corresponds to the median heterozygosity from this study, while those in red below the region names correspond to SSR marker estimates of observed heterozygosity as per Sifres et al. (2011). (*B*) Estimates of pairwise *d*_xy_ and *F*_st_ between SNAPP coalescent clusters. Cells are colored on a continuous scale from blue (high) to pink (low), with yellow/green (intermediate high); orange (intermediate low) colors as intermediate. (*C*) Unrooted tree based on the *d*_xy_ matrix shows differentiation between clusters that follows the two SNAPP superclusters. Branch-wise *d*_xy_ values are shown; *F*_st_ tree shown in supplementary figure S8*A*, Supplementary Material online. (*D*–*F*) Isolation by distance analysis considering all *S. habrochaites* individuals, as well as those in Supercluster 1 and Supercluster 2. Mantel’s test *P*-values were estimated using 100,000 simulated permutations of the Set 2 SNPs.

Heterozygosity was measured separately using just variant or both variant and invariant sites. Both approaches showed that SC accessions had lower heterozygosity than SI accessions (fig. 4*A*, supplementary fig. S7, Supplementary Material online). A previous study (Sifres et al. 2011) concluded based on SSR markers that the highest heterozygosity existed among accessions from the Huancabamba and Cajamarca regions. Our absolute heterozygosity values varied based on whether they were estimated using variant calls or using all sites (see supplementary text 1, Supplementary Material online) and were different from the SSR-based study as expected due to differences in techniques (Sunde et al. 2020). However, the trend between different ecogeographic groups defined using SSRs was consistently reproduced in our analysis (fig. 4*A*;supplementary fig. S7*C*, Supplementary Material online).

Fixation index (*F*_st_) quantifies the degree to which total nucleotide diversity in a pair of populations can be attributed to between-population diversity, with values close to 0 indicating frequent inter-breeding and higher values indicating differentiation (Wright 1931; Weir 2012). Emergence of SC can inflate *F*_st_ values, hence, absolute population divergence (*d*_xy_) (Nei and Li 1979), which ignores within-population diversity, was also calculated. Both *F*_st_ and *d*_xy_ differentiated the two superclusters at the HD (fig. 4*C*, supplementary fig. S8*A*, Supplementary Material online), and showed a close relationship between SI cluster 3 and SC clusters 1 and 2 (fig. 4*B* and *C*, supplementary fig. S8*A*, Supplementary Material online). This could occur either due to continued gene flow or a recent progenitor-descendent relationship, the latter supporting the inference based on reproductive trait analysis (fig. 3*A*, table 1). In the southern part of the range, both metrics also showed a closer genotype sharing between clusters 6–7 and 6–8, but not 7–8 (fig. 4*B*), again providing evidence for a separate establishment of cluster 8 from ancestral populations of clusters 5/6. Interestingly, neighboring SI clusters 4 and 5—located near the HD—were found to be the most differentiated using both *F*_st_ and *d*_xy_ in their pairwise comparisons versus other pairs of neighboring clusters (fig. 4*B* and *C*), mirroring coalescent and split tree analyses. Nucleotide diversity in cluster 4, however, was lower than in clusters 5/6 (supplementary text 1, Supplementary Material online). These observations may result due to a lack of closely related populations among the accessions sampled in clusters 4 and 5 but could also be driven by the geography of the region, where the deep valleys and high mountain ranges near the HD may lead to more reproductive isolation and genetic divergence.

While the AHZ appears to be a diversifying force in the northern and central parts of the range, southern populations are characterized by large geographic distances, creating a new kind of barrier for interpopulation gene exchange, as seen by higher absolute divergence values for clusters 5–8 versus clusters 1–3. Using IBD analysis, we asked if geographical distance played a role in population differentiation. A significant association between genetic and geographic distance was not seen when all the populations were included (association = –0.02, Mantel’s test *P* = 0.77) (fig. 4*D*). When northern and southern superclusters were analyzed separately as superclusters 1 and 2, the northern accessions showed a slight positive yet nonsignificant association of 0.32 (Mantel’s test *P* = 0.06) (fig. 4*E*), but the southern accessions showed a significant IBD with an observed association of 0.63 (Mantel’s test *P* = 0.001) (fig. 4*F*), providing validation for genetic differentiation due to geographical distance.

Overall, these results reveal the role of three important players—the evolution of SC, population fragmentation caused by movement through the AHZ/HD, and IBD south of the HD—in *S. habrochaites* population differentiation. To determine the phenotypic correlates of this population differentiation, we used previously studied metabolic, namely acylsugar, phenotypes as a model.

### Loss of Acylsugar Acetylation Is Associated with Movement through the AHZ and Emergence of SC Groups

**Fig. 5.**
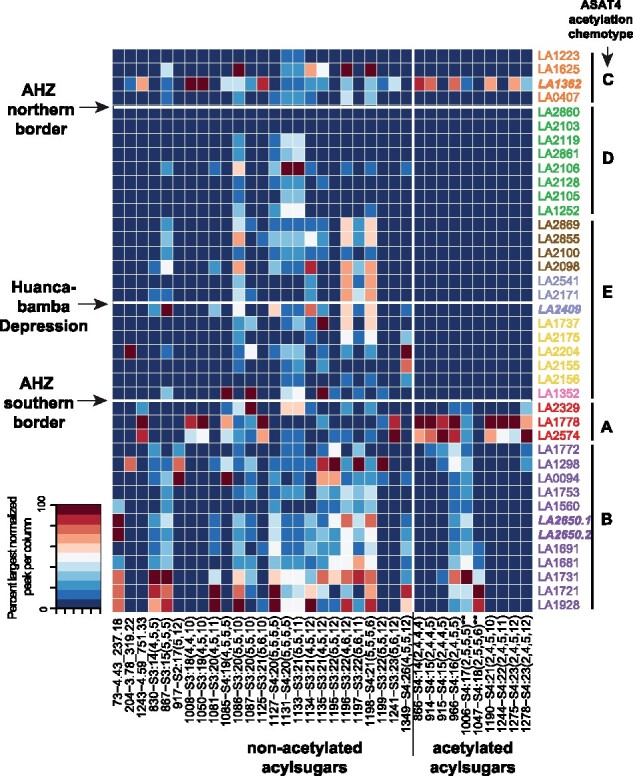
Acylsugar phenotypes across Solanum habrochaites accessions and the genotypes of two associated enzymes. Heatmap of acylsugar peak areas normalized to the internal standard peak area and the maximum area per column. Rows and columns are arranged based on figure 2*A* and types of acylsugars, respectively. Accessions are colored by their population cluster assignments, using color scheme used in figure 2*A*. Three accessions in bold are the geographically misplaced accessions. ASAT4 inactivation chemotypes (*A*–*E*) as per Kim et al. (2012) are also shown. Note that *A*, *B* have acetylated acylsugars and *C*, *D*, *E* contain only nonacetylated acylsugars, due to ASAT4 loss. Column names are in the format (peakID-identified acylsugar). Acylsugars with asterisks indicate those predicted based on MS1 peak and Kim et al. (2012) study without high-confidence MS/MS patterns.

Previous studies identified differences in acylsugar profiles between *S. habrochaites* accessions (Kim et al. 2012; Schilmiller et al. 2015), and here, we asked if these patterns can be explained by genetic structure. Kim et al. (2012) assessed the enzyme ASAT4—the last enzyme in the acylsugar biosynthetic pathway—which was inactivated in many northern accessions resulting in loss of acylsugar acetylation (supplementary fig. S9, Supplementary Material online). We re-analyzed this result in the context of population structure and SC/SI groups. As *S. habrochaites* sampled in this study were obtained from the stock center independently of the previous study, we resampled the leaf surface acylsugars from 2 to 3 replicates of 40 out of 50 accessions used for RAD-seq. In both previous studies (Kim et al. 2012; Schilmiller et al. 2015), LA1362, LA2409, and LA2650 had been identified as chemotypic outliers in their geographical area (i.e., their acylsugar phenotype matched not with their neighbors but with accessions very distant from their documented geographical area). The full RAD-seq data revealed that all three accessions were also genotypic outliers in their recorded geographic area (figs. 1 and 2*A*). However, while LA2409 and LA2650 chemotypes matched previous results and their “true” geographic area based on their genotype, LA1362 profile did not appear as a chemotypic outlier in this study (fig. 5), the reasons for which are not currently clear.

**Fig. 6.**
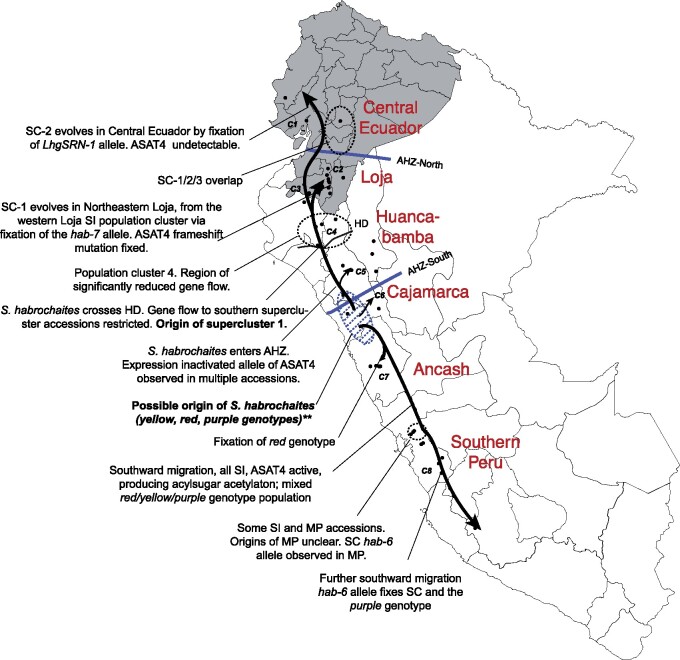
Overall model for *Solanum habrochaites* evolution. This model is based on integrative analysis of the data presented in this paper. Color names noted are as per the colors used in figure 2*A*. Region names refer to the ecogeographic groups of accessions based on Sifres et al. (2011). **The predicted origin region of *S. habrochaites* is also likely the northernmost boundary of its sister species *Solanum pennellii*. Fifty-three out of 56 *S. pennellii* accessions in TGRC are present south of this region.

Loss of acetylation in the north was previously found to occur via three different mechanisms—loss of *ASAT4* gene expression (chemotype E), frameshift mutation in *ASAT4* (chemotype D) and likely loss of the *ASAT4* gene (chemotype C) (Kim et al. 2012). We found that chemotypes C and D were completely confined to the northern SC groups 1–6 (except SC-4, which is in southern Peru), and chemotype E was widespread across SI/MP accessions within the boundary of the AHZ (fig. 5; supplementary fig. S9, Supplementary Material online). The two chemotypes A and B with acetylating, functional versions of ASAT4—representing the ancestral ASAT4 activity—were associated with all SI, MP and SC accessions from clusters 7 and 8. The northernmost accession with a functional *ASAT4* allele (LA2329) lies in cluster 7 in the Cajamarca region south of the AHZ. This finding further supports the model of origination of *S. habrochaites* in the Cajamarca region, and thereby implies that the functional *ASAT4* allele was inactivated during the northward migration into the AHZ (fig. 6).

## Discussion

In this study, we explored the genetic, reproductive and metabolic diversification of *S. habrochaites* in the context of population structure defined using genome-wide RAD-seq markers. Our results show eight different population clusters across the species range, which have been substantially influenced by geographic variation. The Ecuadorian accessions lie in areas that range from densely forested mountains with high precipitation to either dry or wet coastal regions. On the other hand, accessions south of the AHZ in Peru are often confined to isolated river valleys and experience more uniform environments with regard to altitude and temperature fluctuations. Diverse biotic and abiotic interactions and geographical features are associated with a range of metabolic and reproductive phenotypes in this species (Rick et al. 1979; Sacks and St. Clair 1998; Finkers et al. 2007; ten Have et al. 2007; Gonzales-Vigil et al. 2012; Kim et al. 2012; Arms et al. 2015; Schilmiller et al. 2015; Markova et al. 2016; Broz, Randle, et al. 2017; Kilambi et al. 2017; Fan et al. 2017), some of which were assessed here in the context of population history.

Previous studies have placed the origin of *S. habrochaites* in either the Piura-Cajamarca-La Libertad provinces in northern Peru (Rick et al. 1979; Sifres et al. 2011) or slightly more to the south, in the La Libertad-Ancash provinces in north-central Peru (Pease et al. 2016). Based on our population structure analysis (fig. 1*C*), PCA using *S. pennellii* (supplementary fig. S3, Supplementary Material online), as well as a complete lack of the ancestral ASAT4 allele and acetylation phenotypes in northern Peru (fig. 5), we infer that *S. habrochaites* originated in north-central Peru near the southern boundary of the AHZ (fig. 6) supporting the model of Pease et al. (2016). Placing the center of origin in this region also better explains inter-species relationships between *S. habrochaites*–*S. pennellii* given *S. pennellii’s* species range (fig. 6), and with the Arcanum/Esculentum clades of the *Lycopersicon* section of the *Solanum* genus (Pease et al. 2016). After the species originated here, two lineages, representing the two superclusters, then moved north and south from this region to establish the current species range (fig. 6). The eventual populations were likely established through a complex pattern of interpopulation hybridizations, inbreeding and re-establishment (e.g., cluster 7).

Accessions south of the HD are divided into four clusters 5–8. Based on acylsugar phenotypes where *ASAT4* allele is already inactivated in clusters 5 and 6, it is possible that the ancestral population established these clusters *after* clusters 7 and 8—which retain the active/ancestral *ASAT4* allele (fig. 6). Such a topology is also reflected in the coalescent tree (fig. 2*A*). As the species moved further north, it crossed the HD, which is the lowest altitude region in the Peruvian Andes created by the Marañón and Chicama rivers (Weigend 2004). Regional vegetation studies reveal an abrupt shift in vegetation types at the HD (Olson et al. 2001; Richter et al. 2009), illustrative of the geologic history and climatic complexity of the region. These observations have also prompted a debate on whether HD acts as a barrier zone of plant dispersal between Northern and Central Andes (Weigend 2004; Richter et al. 2009; Mutke et al. 2014; Quintana et al. 2017). In our study, a stark contrast is seen in the genotypes spanning the HD (fig. 2*A*). For example, between clusters 4 and 5—which lie geographically close to each other north and south of the HD—both *F*_st_ and *d*_xy_ are higher than between any other pair of neighboring clusters (fig. 4*B*;supplementary fig. S8, Supplementary Material online). Thus, although the HD was clearly not a barrier to *S. habrochaites*’ northward migration, it may exert a dampening effect on gene flow, increasing differentiation between populations north and south of this geological landmark. Identifying the specific nature of evolutionary forces acting on *S. habrochaites* near the HD and generally in the AHZ could help illuminate the reasons for the high floristic diversity in this area.

The northward migration of the species through the AHZ also resulted in fixation of different *S-RNase* and *HT* alleles associated with SC (fig. 3*B*, table 1), likely driven by limiting mates/pollinators. It is noteworthy that the only other tomato clade species on both sides of the AHZ—*S. pimpinellifolium* and *Solanum neorickii—*are SC, suggesting that this mating system may facilitate, or be essential for, successful migration through the fragmented microhabitats in the AHZ. In addition, novel environmental factors, such as new herbivores and/or different temperatures/precipitation levels in the AHZ—a region with high endemism—may have contributed to this evolution.

Our population structure results and reproductive analyses suggest potential progenitor-descendant relationships between SI clusters 5/6 and cluster 8 (SC-4) as well as between SI cluster 3 and clusters 1, 2 (SC-2/3). Given that the known inter-population reproductive barriers are unidirectional, there is still potential for continued gene flow between ancestral SI and derivative SC groups. For example, in the case of the SC-2 and SC-4 groups, the loss of pollen SI factors create a unidirectional inter-population barrier that would only prohibit gene flow between SC males and SI females (Markova et al. 2016; Broz, Randle, et al. 2017). Such unidirectional gene flow between SI and SC populations, as well as hybridization between distinct SC groups, could partially rescue SC populations from the evolutionary “dead end” imposed by the mutational loss of SI (Stebbins 1957; Takebayashi and Morrell 2001; Igic and Busch 2013). The high levels of selfing and the observed interpopulation reproductive barriers could also allow for the purging of deleterious alleles and promote differentiation between populations.

Characterization of the different SC groups in the north also helped explain the observed acylsugar phenotype diversity in this species. *ASAT4* was initially inactivated at the expression level in SI/MP clusters 3–6 during the northward migration (chemotype E; fig. 5). We postulate that this mutation was an epigenetic modification, since the expression of the allele is restored in the SC clusters 1 and 2 accessions, although new deleterious *ASAT4* alleles rose to fixation in those clusters. Prior studies in other Solanaceae species suggest that acyl chains can mediate tritrophic interactions in plant herbivory (Weinhold and Baldwin 2011). However, in this particular case of loss of acetylation, it is not known whether the changes were adaptive and in direct response to the variable habitats of the AHZ, or an indirect consequence of reproductive isolation and genetic differentiation. Nonetheless, in the form of geographic factors and SC, this study adds new evolutionary dimensions to previous research within the broader Solanaceae family that showed multiple factors (e.g., gene duplication, gene loss, enzyme promiscuity, alteration of allosteric regulation, active site substitutions) affecting acylsugar diversity (Kim et al. 2012; Schilmiller et al. 2015; Fan, Miller, et al. 2016; Fan et al. 2017; Moghe et al. 2017; Leong et al. 2020).

In summary, we explored the genetic and phenotypic diversification of *S. habrochaites* across its range using genome-wide SNP analysis. Our results demonstrate the central role played by two geographical features—the AHZ in the north and geographic distance in the south—in establishing reproductively isolated and/or differentiated populations. Evolution of SC in the north has impacted at least one chemical defense phenotype in *S. habrochaites*, and it is possible that both AHZ and SC have influenced the evolution of other traits. Further studies in the AHZ will be crucial in understanding the full extent to which this fascinating geographic feature has impacted the *S. habrochaites* genome and biology. Overall, our findings present a high-resolution view of the microevolutionary processes occurring in *S. habrochaites* and provide greater insights into the molecular pathways generating biodiversity in tropical Andes.

## Materials and Methods

### Plant Growth and Sample Collection for RAD-Seq and Biochemical Analysis

At Michigan State University, 52 accessions of *S. habrochaites* and 4 accessions of *S. pennellii* (LA1941, LA1809, LA1674, LA0716) (supplementary file S1, Supplementary Material online, plus LA2868, LA1978**)** obtained from TGRC were sterilized with 10% trisodium phosphate, germinated on moist filter paper and transferred to peat pots where they were grown for 2 weeks under 16:8 light:dark conditions at 25 °C/16 °C, respectively. Up to four replicates of two-week old plants were then transferred to soil (2 Sure mix + 1/2 sand) where they were grown prior to their harvest for 2 more weeks under the same long day conditions with regular watering. The impact of using stock center accessions on RAD-seq and mating system analysis is described in supplementary text 1, Supplementary Material online. While the TGRC takes substantial efforts to maintain the original genetic diversity in the collections, effects due to uneven temporal sampling as well as genetic bottlenecks due to collecting (generally) 1–5 individuals per location may exist, but were not modeled in our analysis.

### Plant Growth for Reproductive Phenotype Analysis

At Colorado State University, seeds were sterilized according to recommendations of the TGRC (“Tomato Genetics Resource Center”) and were planted into 4-in. pots containing ProMix-BX soil (Premier Tech Horticulture, Quakertown, PA) with 16:8 light:dark conditions 26 °C/18 °C for 2 months. Plants were transplanted to outdoor agricultural fields at Colorado State University (May–September 2017) to obtain sufficient flowers for multiple crosses, and for collection of stylar tissue for immunoblotting analysis. For *S-RNase* allele analysis, plants were grown on a light shelf and a single young leaf was harvested from each plant for DNA preparation as previously described (Broz, Randle, et al. 2017). The impact of using stock center accessions on RAD-seq and mating system analysis is described in supplementary text 1, Supplementary Material online.

### Library Preparation and Sequencing

Leaf tissue from one of the sampled individuals per accession was used for DNA extraction using the Qiagen DNeasy kit (Qiagen, Valencia, CA). Integrity of DNA was verified as a single high molecular weight band on a 1% agarose gel. Biological replicates were obtained for two accessions (LA2098, LA2976 [2×]) and technical replicates for 17 accessions (LA1928, LA1731, LA1778, LA2976, LA1777 [2×], LA2975, LA1986, LA1352, LA2155, LA1737, LA2175, LA2098, LA1252, LA2105, LA2861, LA0407, LA1625 [2×]). Four accessions of *S. pennellii* were selected for outgroup analysis (supplementary file S1, Supplementary Material online)—bringing the total number of RAD-seq samples to 78. One hundred ng of the extracted DNA was used for library preparation and sequencing in two Illumina HiSeq 2000 lanes, as described previously (von Wettberg et al. 2018). Demultiplexed RAD-seq reads were deposited in NCBI Short Read Archive under the BioProject PRJNA623394.

### RAD-Seq Data Processing

Overall, ∼198 million 100 bp single end reads were obtained after standard Illumina quality filtering. We first converted the FASTQ reads from Illumina 1.5 encoding to Sanger encoding using the seqret tool of the EMBOSS v6.5.0 package, trimmed the reads using FASTX toolkit v0.0.14 to a Phred score >20 and selected only 100 bp reads. Since the first base of all reads, which constituted part of the barcode, was “N,” it was trimmed away. The ∼187 million filtered reads were processed using the *process_radtags.pl* script in the Stacks software v2.3d (Rochette et al. 2019) with the following parameter settings *(-b barcodes_6b.tab -q -c -t 90 -E phred33 -D -w 0.20 -s 10 --inline-null -e hindIII --adapter-1 ACACTCTTTCCCTACACGACGCTCTTCCGATCT --adapter-mm 2 --len-limit 90)*. Overall, 85.3% reads passed all quality filtering steps and were deemed high-quality (supplementary file S1, Supplementary Material online). These reads were mapped to the *S. habrochaites* LYC4 genome (Aflitos et al. 2014) using BWA MEM v0.7.17 (Li 2013) with default parameters. Resulting SAM files were converted to BAM and sorted using Samtools v1.9 (Li et al. 2009). Variant calling was performed with Stacks v2.3b using the default parameters for the reference-based mapping pipeline. Unfiltered SNPs were exported using the *populations* module with default parameters. Filtering of SNPs was performed with vcftools v0.1.15 (Danecek et al. 2011) using the following parameters *(--max-missing 0.8 --min-meanDP 6 --max-meanDP 30 --maf 0.05 --mac 3)*. All individuals had <50% missing loci, so none were removed. Heterozygosity and mean read depth were calculated for each sample in vcftools, which resulted in sample LA0716 being removed from downstream analyses due to higher than expected levels of heterozygosity. Given that read depth can influence the accuracy of identifying heterozygotes (Fumagalli 2013), heterozygosity values were calculated in vcftools with several filtering criteria (supplementary text 1, Supplementary Material online). While vcftools only calculates values from variant sites, we wanted to verify the patterns of heterozygosity differences between populations with a different method relying on mapped reads. Therefore, heterozygosity was also calculated in ANGSD (Korneliussen et al. 2014) by only including reads with a high mapping quality using the following parameters *(-dosaf 1 -gl 1 -minQ 20 -minmapq 30*). ANGSD results are shown in the main figure 4*A*.

To make some downstream analyses easier to complete, linkage disequilibrium filtering was performed using Plink v1.90b3.38 and a 10-kb sliding window and a *r*^2^ of 0.2 following a LD decay plot generated in PopLDdecay (Zhang et al. 2019). Two VCF files, the filtered only and filtered with LD pruning, were imported back into Stacks to produce the necessary input files for downstream analyses and calculating *F* statistics (*F*_st_). Summary statistics such F statistics (*F*_st_) and *d*_xy_ were calculated by using the filtered SNP VCF file, as well as directly from the RAD loci in Stacks using the *populations* command (*populations --fstats*). Accession-wise details are provided in supplementary file S1, Supplementary Material online.

### Inference of Population Structure

Population structure was assessed with two different approaches—using inference of ancestral populations and using coalescent analyses. Ancestral population estimation was performed using three different data sets for increased robustness: (Set 1) We assessed 254,263 SNPs using the R package LEA v2.4.0 (Frichot and François 2015). STRUCTURE (Pritchard et al. 2000) and ADMIXTURE (Alexander et al. 2009) rely on simplified population genetic hypotheses, such as the absence of genetic drift, as well as Hardy–Weinberg and linkage equilibrium in ancestral populations. LEA does not rely on the same assumptions and is more appropriate for inbred lineages (Frichot et al. 2014) and was therefore used due to the high-levels of SC found in some populations of *S. habrochaites*. (Set 2) Set 1 was further filtered using LD pruning as described above to produce a total of 93,129 SNPs, and analyzed using LEA. (Set 3). A different run of Stacks was performed using Stacks v1.44, which allowed more granularity in parameter selection. The non-default parameters included *(-T 3 -m 5 -S --bound_low 0 --bound_high 0.02 --alpha 0.05)*. The *populations* module in Stacks v1.44 was called with the following non-default parameters *(-t 3 -r 0.5 -m 5 --min_maf 0.1 --lnl_lim -6 --merge_sites --write_random_snp*). This set contained 25,752 SNPs. For ancestral populations inference, analyses of *K* = 2–15 were performed to determine the best K using the cross-entropy criterion (Frichot et al. 2014), using an alpha value of 100, and 200 iterations. Principal Component Analysis (PCA) as implemented in SNPrelate v1.16.0 (Zheng et al. 2012) was performed using Set 1 and 2 SNPs. Two PCAs were performed for each set: the full data set with all individuals included and with the *S. pennellii* outgroups (LA1674, LA1809, and LA1941) removed.

### Inference of Population Relatedness Using Coalescent Analysis

Coalescent analyses were performed with SNAPP as implemented in BEAST2 v2.4.5 (Bouckaert et al. 2014). Due to the computationally intense nature of SNAPP, the pruned SNP set was further pruned using vcftools by only including sites with no missing data and thinning SNPs to have a minimum of 50,000 bp between successive SNPs. This kept 3,965 SNPs. The resulting VCF file was then converted to a fasta file using vcf2phylip (Ortiz 2019). The XML file was created using BEAUTi keeping each individual as unique species/populations. The mutation rate U and V were calculated from the data set with a coalescence rate set to 10. The MCMC was run for 8 million generations to achieve suitable ESS values (∼449). Tree visualization was performed with DensiTree (part of the Beast package) using a 25% burnin. The maximum clade credibility (MCC) was also identified with TreeAnnotator (part of the Beast package) using a 25% burnin. The tree allowed for classification of eight clades within the ingroup (plus the outgroup). These clades were then used for downstream analyses that required population specification. The MCC was then plotted on a map along with heterozygosity levels using the R package phytools v0.6.99 (Revell 2012).

### Other Population Genomic Analyses

Split network analysis was performed by implementing a Neighbor Net within splitsTree4 (Huson and Bryant 2006). The program was run with default parameters using the same set of 3,965 SNPs used for coalescent analysis. IBD analyses were performed using Mantel’s test of correlation between the genetic and geographic distance matrices using Set 2 SNPs. The genepop file produced by Stacks was read into the R package adegenet v2.1.1 (Jombart 2008; Jombart and Ahmed 2011) as each accession being a unique population. Two sets of analyses were done: one with all the accessions found in the coalescent analysis (minus the outgroup) and a second set of analyses with samples split between northern and southern super-clusters as seen with the MCC plotted on a map. Both analyses used Euclidean distances and 100,000 permutations.

### TSS for Population Structure

Twenty-two broadly represented high coverage loci were identified for TSS (supplementary text 1, Supplementary Material online). An initial nucleotide BLAST was performed using the *LYC4* (Aflitos et al. 2014) *S. habrochaites* assembly to identify sequences surrounding the polymorphic sequences for each of the 22 loci. Primers were designed to specifically amplify ∼200 bp spanning the polymorphic regions of each locus, and the resulting PCR products were purified (Zymo, Irvine, CA), and sequenced (GENEWIZ, South Plainfield, NJ). For each of the 22 loci, sequences were aligned in MEGA7 (Kumar et al. 2016) using Muscle (Edgar 2004) with the original intended target, the corresponding sequence of LA0407 from the original RAD-seq data set, and the top BLAST hits.

The diploid state of each locus of each accession was determined by aligning the sequences for all accessions, trimming off poor-quality sequences, and examining the set of trace files for each locus manually for heterozygous base calls (Chromas Pro, https://technelysium.com.au/wp/chromaspro/, last accessed April 01, 2021). If no ambiguous calls were present in the trace files, the individual was assumed to be homozygous at that locus.

To combine TSS and RAD-seq data, we performed BLAST between the trimmed Sanger sequences and the set of all 22 RAD loci consensus sequences to identify each corresponding RAD-seq locus. Sequences representing the 22 loci were extracted from the RAD-seq data (51 samples) in the *populations* module of STACKS v1.46 using a selection of loci identified by BLAST. These sequences were combined with their TSS counterparts using custom scripts, aligned, manually inspected, and trimmed when necessary. PGDSpider v2.1.1.2 (Lischer and Excoffier 2012) was used to call allele variants for each locus; the separate matrices generated by PGDSpider were then combined to create the STRUCTURE v2.3.4 (Pritchard et al. 2000) input matrix of allele variants for all 22 loci for the 69 total samples (15 Sanger sequences from accessions not in the original experiment, 3 Sanger sequences from accessions included in the original RAD-seq data set, and 51 sequences from the RAD-seq samples) using a custom Python script. STRUCTURE was run using default parameters with no prior population groups assumed for *K* = 1–8 (three replicates per *K*) for 10,000 burn-in and 10,000 MCMC cycles. Results were extracted using STRUCTURE HARVESTER vA.2 July 2014 (Earl and vonHoldt 2012) and replicate runs were combined using CLUMPP v1.1 (Jakobsson and Rosenberg 2007). All statistics (adegenet v1.7-15, pophelper v2.3.0), data analysis (pophelper), and plot generation (ggplot2, scatterpie) were performed using R v3.4.1 (Jombart and Ahmed 2011; Francis 2017).

### Identification of the *hab-7* Allele

A previously published stylar transcriptome of SC accession LA2119 (Broz, Guerrero, et al. 2017) was used as a BLAST database to discover potential *S-RNase* alleles (NCBI BioProject PRJNA310635). Using a set of known *S-RNase* gene sequences as BlastN queries to the LA2119 assembly, a single putative *S-RNase* transcript sequence was recovered. Allele-specific primers were designed using this putative *S-RNas*e sequence (supplementary file S2, Supplementary Material online), and PCR was performed using genomic DNA from multiple LA2119 individuals. Amplicon sequencing verified the sequence identified by the transcriptome analysis and revealed the presence of a single intron in genomic DNA. Following the convention set by Covey et al. (2010), this S-RNase allele was dubbed *hab-7*. The transcript abundances of the *hab-7* allele in LA2119 styles and two different *S-RNase* alleles of SI *S. habrochaites* accession LA1777 were identified using data from a previous *S. habrochaites* transcriptome study (Broz, Guerrero, et al. 2017).

### Reproductive Trait Analysis

At least two individuals (each grown from a separate seed) of each accession were used for phenotyping. Mating system was determined for previously untested northern accessions (supplementary fig. S5, Supplementary Material online) and verified in an additional set of accessions using self-pollinations as previously described (Broz, Randle, et al. 2017). If production of self-fruit was observed, plants were recorded as SC. If plants failed to set self-fruit using this approach, hand pollinations were performed, and/or pollen tube growth in styles was assessed as previously described (Covey et al. 2010). When at least three pollen tubes could be visualized at the base of the style or in the ovary in multiple independent crosses, plants were considered SC. When no self-fruit was formed and pollen tube tips could clearly be visualized terminating within the style, plants were considered SI.

To test for inter-population reproductive barriers as initially described by Martin (1961), hand pollinations were performed using *S. habrochaites* SC accession LA0407 (SC-2 group) as male to test for the presence of pistil barriers that reject pollen of SC-2 plants and SI accession LA1777 as female to test for pollen resistance to S-RNase barriers as previously described (Broz, Randle, et al. 2017). To test for interspecific reproductive barriers, pistils of *S. habrochaites* accessions were pollinated using *Solanum lycopersicum* cultivars VF36, M82 or LA1221 as males.

Expression of S-RNase and an additional pistil SI factor, HT-protein, was assessed in stylar extracts from at least two individuals using immunoblotting with anti-peptide antibodies specific to each protein as described previously (Covey et al. 2010; Chalivendra et al. 2013; Broz, Randle, et al. 2017). The presence or absence of specific *S-RNase* alleles was determined for at least three individuals from each accession. *S-RNase* alleles were amplified from genomic DNA of individual plants using allele-specific primers (supplementary file S2, Supplementary Material online) in PCR reactions, as described previously (Broz, Randle, et al. 2017). In selected accessions, the *HT* gene was amplified from genomic DNA using conserved gene specific primers (Covey et al. 2010), PCR products were purified and subjected to Sanger sequencing.

### Acylsugar Sampling and MS Data Analysis

Acylsugar sampling was performed from a single uniformly sized young leaflet of 2–3 plants per accession for 40 of the 50 accessions as previously described (Fan, Moghe, et al. 2016). Metabolites were analyzed on a Supelco Ascentis C18 column, using a Shimadzu Ultra High Performance Liquid Chromatograph (UHPLC) connected to a Waters Xevo quadrupole Time of Flight mass spectrometer (MS). Raw files were converted into ABF format using Reifycs Abf Converter (https://www.reifycs.com/AbfConverter/, last accessed April 01, 2021) and imported into MS-DIAL (Tsugawa et al. 2015) for preprocessing. Peaks with an amplitude >100 and with >2 data points were considered for further analysis. Mass slice width and sigma windows were set to 0.05 and 0.5, respectively. Peaks across all samples were aligned with a 0.05 min. retention time tolerance and 0.03 Da MS1 tolerance. Acylsugars were then selected and annotated from the alignment results based on manually identified acylsugar peaks, MS1 *m/z* values, MS/MS and previous results (Kim et al. 2012). A 5-fold sample max/blank average filter was applied across the samples. Normalized and filtered data were then exported. Extracted peak areas were normalized by the internal standard peak areas per sample, and the normalized peak areas were averaged for each accession. Only peaks >2× internal standard peak area in >2 accessions were considered reliable signals.

## Supplementary Material

Supplementary data are available at *Molecular Biology and Evolution* online.

## Supplementary Material

msab092_Supplementary_Data

## References

[msab092-B1] Aflitos S , Schijlen E, de Jong H, de Ridder D, Smit S, Finkers R, Wang J, Zhang G, Li N, Mao L, et al 2014. Exploring genetic variation in the tomato (Solanum section Lycopersicon) clade by whole-genome sequencing. Plant J. 80(1):136–148. 25039268 10.1111/tpj.12616

[msab092-B2] Alexander DH , Novembre J, Lange K. 2009. Fast model-based estimation of ancestry in unrelated individuals. Genome Res. 19(9):1655–1664. 19648217 10.1101/gr.094052.109 PMC2752134

[msab092-B3] Andrews KR , Good JM, Miller MR, Luikart G, Hohenlohe PA. 2016. Harnessing the power of RADseq for ecological and evolutionary genomics. Nat Rev Genet. 17(2):81–92. 26729255 10.1038/nrg.2015.28 PMC4823021

[msab092-B4] Antonelli A , Nylander JAA, Persson C, Sanmartín I. 2009. Tracing the impact of the Andean uplift on Neotropical plant evolution. Proc Natl Acad Sci USA. 106(24):9749–9754. 19470489 10.1073/pnas.0811421106 PMC2685738

[msab092-B5] Arms EM , Bloom AJ, St. Clair DA. 2015. High-resolution mapping of a major effect QTL from wild tomato *Solanum habrochaites* that influences water relations under root chilling. Theor Appl Genet. 128(9):1713–1724. 26044122 10.1007/s00122-015-2540-y PMC4540768

[msab092-B6] Baek YS , Covey PA, Petersen JJ, Chetelat RT, McClure B, Bedinger PA. 2015. Testing the SI × SC rule: pollen–pistil interactions in interspecific crosses between members of the tomato clade (Solanum section Lycopersicon, Solanaceae). Am J Bot. 102(2):302–311. 25667082 10.3732/ajb.1400484

[msab092-B7] Baird NA , Etter PD, Atwood TS, Currey MC, Shiver AL, Lewis ZA, Selker EU, Cresko WA, Johnson EA. 2008. Rapid SNP discovery and genetic mapping using sequenced RAD markers. PLoS One 3(10):e3376. 18852878 10.1371/journal.pone.0003376 PMC2557064

[msab092-B8] Baker HG. 1955. Self-compatibility and establishment after “long-distance” dispersal. Evolution 9(3):347–349.

[msab092-B9] Baker HG. 1967. Support for Baker’s law—as a rule. Evolution 21(4):853–856. 28563079 10.1111/j.1558-5646.1967.tb03440.x

[msab092-B10] Bedinger PA , Broz AK, Tovar-Mendez A, McClure B. 2017. Pollen–pistil interactions and their role in mate selection. Plant Physiol. 173(1):79–90. 27899537 10.1104/pp.16.01286 PMC5210727

[msab092-B11] Bedinger PA , Chetelat RT, McClure B, Moyle LC, Rose JKC, Stack SM, van der Knaap E, Baek YS, Lopez-Casado G, Covey PA, et al 2011. Interspecific reproductive barriers in the tomato clade: opportunities to decipher mechanisms of reproductive isolation. Sex Plant Reprod. 24(3):171–187. 21076968 10.1007/s00497-010-0155-7

[msab092-B12] Berry PE. 1982. The systematics and evolution of fuchsia sect. Fuchsia (onagraceae). Ann Missouri Bot Gard. 69(1):1–198.

[msab092-B13] Bouckaert R , Heled J, Kühnert D, Vaughan T, Wu C-H, Xie D, Suchard MA, Rambaut A, Drummond AJ. 2014. BEAST 2: a software platform for Bayesian evolutionary analysis. PLoS Comput Biol. 10(4):e1003537. 24722319 10.1371/journal.pcbi.1003537 PMC3985171

[msab092-B14] Broz AK , Guerrero RF, Randle AM, Baek YS, Hahn MW, Bedinger PA. 2017. Transcriptomic analysis links gene expression to unilateral pollen-pistil reproductive barriers. BMC Plant Biol. 17(1):81. 28438120 10.1186/s12870-017-1032-4 PMC5402651

[msab092-B15] Broz AK , Randle AM, Sianta SA, Tovar-Méndez A, McClure B, Bedinger PA. 2017. Mating system transitions in *Solanum habrochaites* impact interactions between populations and species. New Phytol. 213(1):440–454. 27516156 10.1111/nph.14130

[msab092-B16] Bryant D , Bouckaert R, Felsenstein J, Rosenberg NA, RoyChoudhury A. 2012. Inferring species trees directly from biallelic genetic markers: bypassing gene trees in a full coalescent analysis. Mol Biol Evol. 29(8):1917–1932. 22422763 10.1093/molbev/mss086 PMC3408069

[msab092-B17] Catchen J , Hohenlohe PA, Bassham S, Amores A, Cresko WA. 2013. Stacks: an analysis tool set for population genomics. Mol Ecol. 22(11):3124–3140. 23701397 10.1111/mec.12354 PMC3936987

[msab092-B18] Chalivendra SC , Lopez-Casado G, Kumar A, Kassenbrock AR, Royer S, Tovar-Mèndez A, Covey PA, Dempsey LA, Randle AM, Stack SM, et al 2013. Developmental onset of reproductive barriers and associated proteome changes in stigma/styles of *Solanum pennellii*. J Exp Bot. 64(1):265–279. 23166371 10.1093/jxb/ers324 PMC3528032

[msab092-B19] Covey PA , Kondo K, Welch L, Frank E, Sianta S, Kumar A, Nuñez R, Lopez‐Casado G, Knaap EVD, Rose JKC, et al 2010. Multiple features that distinguish unilateral incongruity and self-incompatibility in the tomato clade. Plant J. 64(3):367–378. 20804455 10.1111/j.1365-313X.2010.04340.x

[msab092-B20] Danecek P , Auton A, Abecasis G, Albers CA, Banks E, DePristo MA, Handsaker RE, Lunter G, Marth GT, Sherry ST, et al 2011. The variant call format and VCFtools. Bioinformatics 27(15):2156–2158. 21653522 10.1093/bioinformatics/btr330 PMC3137218

[msab092-B21] Darwin C. 1859. On the origin of the species by means of natural selection: or, the preservation of favoured races in the struggle for life. London: John Murray. PMC5184128 30164232

[msab092-B6185529] Earl DA, , vonHoldt BM. 2012. STRUCTURE HARVESTER: a website and program for visualizing STRUCTURE output and implementing the Evanno method. Conserv Genet Resour. 4:359–361.

[msab092-B22] Edgar RC. 2004. MUSCLE: multiple sequence alignment with high accuracy and high throughput. Nucleic Acids Res. 32(5):1792–1797. 15034147 10.1093/nar/gkh340 PMC390337

[msab092-B23] Fan P , Miller AM, Liu X, Jones AD, Last RL. 2017. Evolution of a flipped pathway creates metabolic innovation in tomato trichomes through BAHD enzyme promiscuity. Nat Commun. 8(1):2080. 29234041 10.1038/s41467-017-02045-7 PMC5727100

[msab092-B24] Fan P , Miller AM, Schilmiller AL, Liu X, Ofner I, Jones AD, Zamir D, Last RL. 2016. *In vitro* reconstruction and analysis of evolutionary variation of the tomato acylsucrose metabolic network. Proc Natl Acad Sci USA. 113(2):E239–248. 26715757 10.1073/pnas.1517930113 PMC4720351

[msab092-B25] Fan P , Moghe GD, Last RL. 2016. Comparative biochemistry and in vitro pathway reconstruction as powerful partners in studies of metabolic diversity. In: O’Connor SE, editor. Synthetic biology and metabolic engineering in plants and microbes. Part B: metabolism in plants. Methods in enzymology. Massachusetts: Academic Press. p. 1–17. 10.1016/bs.mie.2016.02.023 27480680

[msab092-B26] Finkers R , van Heusden AW, Meijer-Dekens F, van Kan JAL, Maris P, Lindhout P. 2007. The construction of a *Solanum habrochaites* LYC4 introgression line population and the identification of QTLs for resistance to Botrytis cinerea. Theor Appl Genet. 114(6):1071–1080. 17273845 10.1007/s00122-006-0500-2 PMC1913174

[msab092-B27] Francis RM. 2017. pophelper: an R package and web app to analyse and visualize population structure. Mol Ecol Resour. 17(1):27–32. 26850166 10.1111/1755-0998.12509

[msab092-B28] Frichot E , François O. 2015. LEA: an R package for landscape and ecological association studies. Methods Ecol Evol. 6(8):925–929.

[msab092-B29] Frichot E , Mathieu F, Trouillon T, Bouchard G, François O. 2014. Fast and efficient estimation of individual ancestry coefficients. Genetics 196(4):973–983. 24496008 10.1534/genetics.113.160572 PMC3982712

[msab092-B30] Fumagalli M. 2013. Assessing the effect of sequencing depth and sample size in population genetics inferences. PLoS One 8(11):e79667. 24260275 10.1371/journal.pone.0079667 PMC3832539

[msab092-B31] Gonzales-Vigil E , Hufnagel DE, Kim J, Last RL, Barry CS. 2012. Evolution of TPS20-related terpene synthases influences chemical diversity in the glandular trichomes of the wild tomato relative *Solanum habrochaites*. Plant J. 71(6):921–935. 22563774 10.1111/j.1365-313X.2012.05040.x PMC3466413

[msab092-B32] Harvey MG , Singhal S, Rabosky DL. 2019. Beyond reproductive isolation: demographic controls on the speciation process. Annu Rev Ecol Evol Syst. 50(1):75–95.

[msab092-B33] ten Have A , van Berloo R, Lindhout P, van Kan JAL. 2007. Partial stem and leaf resistance against the fungal pathogen Botrytis cinerea in wild relatives of tomato. Eur J Plant Pathol. 117(2):153–166.

[msab092-B34] Hazzi NA , Moreno JS, Ortiz-Movliav C, Palacio RD. 2018. Biogeographic regions and events of isolation and diversification of the endemic biota of the tropical Andes. Proc Natl Acad Sci USA. 115(31):7985–7990. 30018064 10.1073/pnas.1803908115 PMC6077705

[msab092-B35] Hoorn C , Wesselingh FP, Steege H. T, Bermudez MA, Mora A, Sevink J, Sanmartín I, Sanchez-Meseguer A, Anderson CL, Figueiredo JP, et al 2010. Amazonia through time: Andean uplift, climate change, landscape evolution, and biodiversity. Science 330(6006):927–931. 21071659 10.1126/science.1194585

[msab092-B36] Huson DH , Bryant D. 2006. Application of phylogenetic networks in evolutionary studies. Mol Biol Evol. 23(2):254–267. 16221896 10.1093/molbev/msj030

[msab092-B37] Igic B , Busch JW. 2013. Is self-fertilization an evolutionary dead end? New Phytol. 198(2):386–397. 23421594 10.1111/nph.12182

[msab092-B38] Igic B , Lande R, Kohn JR. 2008. Loss of self‐incompatibility and its evolutionary consequences. Int J Plant Sci. 169(1):93–104.

[msab092-B39] Jakobsson M , Rosenberg NA. 2007. CLUMPP: a cluster matching and permutation program for dealing with label switching and multimodality in analysis of population structure. Bioinformatics (Oxf, Engl). 23(14):1801–1806. 10.1093/bioinformatics/btm233 17485429

[msab092-B40] Jombart T. 2008. adegenet: a R package for the multivariate analysis of genetic markers. Bioinformatics (Oxf, Engl). 24(11):1403–1405. 10.1093/bioinformatics/btn129 18397895

[msab092-B41] Jombart T , Ahmed I. 2011. adegenet 1.3-1: new tools for the analysis of genome-wide SNP data. Bioinformatics (Oxf, Engl). 27(21):3070–3071. 10.1093/bioinformatics/btr521 PMC3198581 21926124

[msab092-B42] Kilambi HV , Manda K, Rai A, Charakana C, Bagri J, Sharma R, Sreelakshmi Y. 2017. Green-fruited *Solanum habrochaites* lacks fruit-specific carotenogenesis due to metabolic and structural blocks. J Exp Bot. 68(17):4803–4819. 29048567 10.1093/jxb/erx288 PMC5853803

[msab092-B43] Kim J , Kang K, Gonzales-Vigil E, Shi F, Jones AD, Barry CS, Last RL. 2012. Striking natural diversity in glandular trichome acylsugar composition is shaped by variation at the Acyltransferase2 locus in the wild tomato *Solanum habrochaites*. Plant Physiol. 160(4):1854–1870. 23054567 10.1104/pp.112.204735 PMC3510116

[msab092-B44] Knapp S , Spooner DM. 1999. A new name for a common Ecuadorian and Peruvian wild tomato species. Novon 9(3):375–376.

[msab092-B45] Kondo K , Yamamoto M, Itahashi R, Sato T, Egashira H, Hattori T, Kowyama Y. 2002. Insights into the evolution of self-compatibility in Lycopersicon from a study of stylar factors. Plant J. 30(2):143–153. 12000451 10.1046/j.1365-313x.2002.01275.x

[msab092-B46] Korneliussen TS , Albrechtsen A, Nielsen R. 2014. ANGSD: analysis of next generation sequencing data. BMC Bioinformatics 15:356. 25420514 10.1186/s12859-014-0356-4 PMC4248462

[msab092-B47] Kumar S , Stecher G, Tamura K. 2016. MEGA7: molecular evolutionary genetics analysis version 7.0 for bigger datasets. Mol Biol Evol. 33(7):1870–1874. 27004904 10.1093/molbev/msw054 PMC8210823

[msab092-B48] Lande R , Schemske DW. 1985. The evolution of self-fertilization and inbreeding depression in plants. I. Genetic models. Evolution 39(1):24–40. 28563655 10.1111/j.1558-5646.1985.tb04077.x

[msab092-B49] Leckie BM , D'Ambrosio DA, Chappell TM, Halitschke R, De Jong DM, Kessler A, Kennedy GG, Mutschler MA. 2016. Differential and synergistic functionality of acylsugars in suppressing oviposition by insect herbivores. PLoS One 11(4):e0153345. 27065236 10.1371/journal.pone.0153345 PMC4827819

[msab092-B50] Leong BJ , Hurney SM, Fiesel PD, Moghe GD, Jones AD, Last RL. 2020. Specialized metabolism in a nonmodel nightshade: trichome acylinositol biosynthesis. Plant Physiol. 183(3):915–924. 32354879 10.1104/pp.20.00276 PMC7333698

[msab092-B51] Li H. 2013. Aligning sequence reads, clone sequences and assembly contigs with BWA-MEM. *arXiv:1303.3997 [q-bio]*.

[msab092-B52] Li H , Handsaker B, Wysoker A, Fennell T, Ruan J, Homer N, Marth G, Abecasis G, Durbin R, 2009. Genome project data processing subgroup. 2009. The sequence alignment/map format and SAMtools. Bioinformatics (Oxf, Engl). 25(16):2078–2079. 10.1093/bioinformatics/btp352 PMC2723002 19505943

[msab092-B53] Lischer HEL , Excoffier L. 2012. PGDSpider: an automated data conversion tool for connecting population genetics and genomics programs. Bioinformatics 28(2):298–299. 22110245 10.1093/bioinformatics/btr642

[msab092-B54] Markova DN , Petersen JJ, Qin X, Short DR, Valle MJ, Tovar-Méndez A, McClure BA, Chetelat RT. 2016. Mutations in two pollen self-incompatibility factors in geographically marginal populations of *Solanum habrochaites* impact mating system transitions and reproductive isolation. Am J Bot. 103(10):1847–1861. 27793860 10.3732/ajb.1600208

[msab092-B55] Martin FW. 1961. Complex hnilateral hybridization in *Lycopersicon hirsutum*. Proc Natl Acad Sci USA. 47:855–857. 13767338 10.1073/pnas.47.6.855 PMC221351

[msab092-B56] Martin FW. 1963. Distribution and interrelationships of incompatibility barriers in the Lycopersicon Hirsutum Humb. and Bonpl. complex. Evolution 17(4):519–528.

[msab092-B57] Mayr E. 1942. Systematics and the origin of species from the viewpoint of a zoologist. New York: Columbia University Press.

[msab092-B58] McClure B , Mou B, Canevascini S, Bernatzky R. 1999. A small asparagine-rich protein required for S-allele-specific pollen rejection in Nicotiana. Proc Natl Acad Sci USA. 96(23):13548–13553. 10557358 10.1073/pnas.96.23.13548 PMC23985

[msab092-B59] Miller MR , Dunham JP, Amores A, Cresko WA, Johnson EA. 2007. Rapid and cost-effective polymorphism identification and genotyping using restriction site associated DNA (RAD) markers. Genome Res. 17(2):240–248. 17189378 10.1101/gr.5681207 PMC1781356

[msab092-B60] Moghe GD , Leong BJ, Hurney S, Jones AD, Last RL. 2017. Evolutionary routes to biochemical innovation revealed by integrative analysis of a plant-defense related specialized metabolic pathway. eLife6:e28468. 10.7554/eLife.28468 PMC5595436 28853706

[msab092-B61] Mutke J , Jacobs R, Meyers K, Henning T, Weigend M. 2014. Diversity patterns of selected Andean plant groups correspond to topography and habitat dynamics, not orogeny. Front Genet. 5: 351. 25346750 10.3389/fgene.2014.00351 PMC4193334

[msab092-B62] Mutschler MA , Liedl BE. 1994. Interspecific crossing barriers in Lycopersicon and their relationship to self-incompatibility. In: Williams EG, Clarke AE, Knox RB, editors. Advances in cellular and molecular biology of plants. Genetic control of self-incompatibility and reproductive development in flowering plants. Dordrecht (Netherlands): Springer. p. 164–188.

[msab092-B63] Myers N , Mittermeier RA, Mittermeier CG, da Fonseca GAB, Kent J. 2000. Biodiversity hotspots for conservation priorities. Nature 403(6772):853–858. 10706275 10.1038/35002501

[msab092-B64] Nei M , Li WH. 1979. Mathematical model for studying genetic variation in terms of restriction endonucleases. Proc Natl Acad Sci USA. 76(10):5269–5273. 291943 10.1073/pnas.76.10.5269 PMC413122

[msab092-B65] Olson DM , Dinerstein E, Wikramanayake ED, Burgess ND, Powell GVN, Underwood EC, D'amico JA, Itoua I, Strand HE, Morrison JC, et al 2001. Terrestrial ecoregions of the world: a new map of life on earth. BioScience 51(11):933–938.

[msab092-B66] Ortiz E. 2019. *vcf2phylip v2.0: convert a VCF matrix into several matrix formats for phylogenetic analysis*. Zenodo.

[msab092-B67] Pannell JR , Auld JR, Brandvain Y, Burd M, Busch JW, Cheptou P-O, Conner JK, Goldberg EE, Grant A-G, Grossenbacher DL, et al 2015. The scope of Baker’s law. New Phytol. 208(3):656–667. 26192018 10.1111/nph.13539

[msab092-B68] Pease JB , Haak DC, Hahn MW, Moyle LC. 2016. Phylogenomics reveals three sources of adaptive variation during a rapid radiation. PLoS Biol. 14(2):e1002379. 26871574 10.1371/journal.pbio.1002379 PMC4752443

[msab092-B69] Peralta IE , Spooner DM, Knapp S. 2008. Taxonomy of wild tomatoes and their relatives (Solanum sect. Lycopersicoides, sect. Juglandifolia, sect. Lycopersicon; Solanaceae). Syst Bot Monogr. 84: 1–186.

[msab092-B70] Pritchard JK , Stephens M, Donnelly P. 2000. Inference of population structure using multilocus genotype data. Genetics 155(2):945–959. 10835412 10.1093/genetics/155.2.945 PMC1461096

[msab092-B71] Quintana C , Pennington RT, Ulloa CU, Balslev H. 2017. Biogeographic barriers in the Andes: is the Amotape—Huancabamba zone a dispersal barrier for dry forest plants? Annals Missouri Bot Gard. 102(3):542–550.

[msab092-B72] Revell LJ. 2012. phytools: an R package for phylogenetic comparative biology (and other things). Methods Ecol Evol. 3(2):217–223.

[msab092-B73] Reznick DN , Ricklefs RE. 2009. Darwin’s bridge between microevolution and macroevolution. Nature 457(7231):837–842. 19212402 10.1038/nature07894

[msab092-B74] Richter M , Diertl K-H, Emck P, Thorsten P, Beck E. 2009. Reasons for an outstanding plant diversity in the tropical Andes of Southern Ecuador. Landscape Online 12:1–6.

[msab092-B75] Rick C , Chetelat R. 1991. The breakdown of self‐incompatibility in *Lycopersicon hirsutum*. In: Hawkes L, Nee M, Estrada N, editors. Solanaceae III: taxonomy, chemistry, evolution. London (United Kingdom): Royal Bothanic Gardens Kew and Linnean Society of London. p. 253–256.

[msab092-B76] Rick CM , Fobes JF, Tanksley SD. 1979. Evolution of mating systems in *Lycopersicon hirsutum* as deduced from genetic variation in electrophoretic and morphological characters. Plant Syst Evol. 132(4):279–298.

[msab092-B77] Rochette NC , Rivera‐Colón AG, Catchen JM. 2019. Stacks 2: analytical methods for paired-end sequencing improve RADseq-based population genomics. Mol Ecol. 28(21):4737–4754. 31550391 10.1111/mec.15253

[msab092-B78] Sacks EJ , St. Clair DA. 1998. Variation among seven genotypes of *Lycopersicon esculentum* and 36 accessions of *L. hirsutum* for interspecific crossability. Euphytica 101(2):185–191.

[msab092-B79] Schemske DW , Lande R. 1985. The evolution of self-fertilization and inbreeding depression in plants. II. Empirical observations. Evolution 39(1):41–52. 28563649 10.1111/j.1558-5646.1985.tb04078.x

[msab092-B80] Schilmiller AL , Moghe GD, Fan P, Ghosh B, Ning J, Jones AD, Last RL. 2015. Functionally divergent alleles and duplicated loci encoding an acyltransferase contribute to acylsugar metabolite diversity in *Solanum* trichomes. Plant Cell. 27(4):1002–1017. 25862303 10.1105/tpc.15.00087 PMC4558703

[msab092-B81] Schlichting CD. 1986. The evolution of phenotypic plasticity in plants. Annu Rev Ecol Syst. 17(1):667–693.

[msab092-B82] Sifres A , Blanca J, Nuez F. 2011. Pattern of genetic variability of *Solanum habrochaites* in its natural area of distribution. Genet Resour Crop Evol. 58(3):347–360.

[msab092-B83] Stebbins GL. 1957. Self fertilization and population variability in the higher plants. Am Nat. 91(861):337–354.

[msab092-B84] Stebbins GL. 1974. Flowering plants: evolution above the species level. Massachusetts: Harvard University Press.

[msab092-B85] Sunde J , Yıldırım Y, Tibblin P, Forsman A. 2020. Comparing the performance of microsatellites and RADseq in population genetic studies: analysis of data for pike (*Esox lucius*) and a synthesis of previous studies. Front Genet. 11:218. 32231687 10.3389/fgene.2020.00218 PMC7082332

[msab092-B86] Takayama S , Isogai A. 2005. Self-incompatibility in plants. Annu Rev Plant Biol. 56:467–489. 15862104 10.1146/annurev.arplant.56.032604.144249

[msab092-B87] Takebayashi N , Morrell PL. 2001. Is self-fertilization an evolutionary dead end? Revisiting an old hypothesis with genetic theories and a macroevolutionary approach. Am J Bot. 88(7):1143–1150. 11454614

[msab092-B88] Tomato Genetics Resource Center. Available from: https://tgrc.ucdavis.edu/. Accessed April 1, 2021.

[msab092-B89] Tovar‐Méndez A , Kumar A, Kondo K, Ashford A, Baek YS, Welch L, Bedinger PA, McClure BA. 2014. Restoring pistil-side self-incompatibility factors recapitulates an interspecific reproductive barrier between tomato species. Plant J. 77(5):727–736. 24387692 10.1111/tpj.12424

[msab092-B90] Tovar-Méndez A , Lu L, McClure B. 2017. HT proteins contribute to S-RNase-independent pollen rejection in Solanum. Plant J. 89(4):718–729. 27862494 10.1111/tpj.13416

[msab092-B91] Tsugawa H , Cajka T, Kind T, Ma Y, Higgins B, Ikeda K, Kanazawa M, VanderGheynst J, Fiehn O, Arita M. 2015. MS-DIAL: data-independent MS/MS deconvolution for comprehensive metabolome analysis. Nat Methods. 12(6):523–526. 25938372 10.1038/nmeth.3393 PMC4449330

[msab092-B92] Weigend M. 2002. Observations on the biogeography of the Amotape-Huancabamba Zone in Northern Peru. Bot Rev. 68(1):38–54.

[msab092-B93] Weigend M. 2004. Additional observations on the biogeography of the Amotape-Huancabamba zone in Northern Peru: defining the South-Eastern limits. Rev Peru Biol. 11:127–134.

[msab092-B94] Weinhold A , Baldwin IT. 2011. Trichome-derived *O*-acyl sugars are a first meal for caterpillars that tags them for predation. Proc Natl Acad Sci USA. 108(19):7855–7859. 21518882 10.1073/pnas.1101306108 PMC3093468

[msab092-B95] Weir BS. 2012. Estimating F-statistics: a historical view. Philos Sci. 79(5):637–643. 26405363 10.1086/667904 PMC4578636

[msab092-B96] von Wettberg EJB , Chang PL, Başdemir F, Carrasquila-Garcia N, Korbu LB, Moenga SM, Bedada G, Greenlon A, Moriuchi KS, Singh V, et al 2018. Ecology and genomics of an important crop wild relative as a prelude to agricultural innovation. Nat Commun. 9(1):649. 29440741 10.1038/s41467-018-02867-z PMC5811434

[msab092-B97] Wright S. 1931. Evolution in Mendelian populations. Genetics 16(2):97–159. 17246615 10.1093/genetics/16.2.97 PMC1201091

[msab092-B98] Zhang C , Dong S-S, Xu J-Y, He W-M, Yang T-L. 2019. PopLDdecay: a fast and effective tool for linkage disequilibrium decay analysis based on variant call format files. Bioinformatics (Oxf, Engl). 35(10):1786–1788. 10.1093/bioinformatics/bty875 30321304

[msab092-B99] Zheng X , Levine D, Shen J, Gogarten SM, Laurie C, Weir BS. 2012. A high-performance computing toolset for relatedness and principal component analysis of SNP data. Bioinformatics (Oxf, Engl). 28(24):3326–3328. 10.1093/bioinformatics/bts606 PMC3519454 23060615

