# Supplementary material for: Migration through a Major Andean Ecogeographic Disruption as a Driver of Genetic and Phenotypic Diversity in a Wild Tomato Species
Source: Mol Biol Evol. 2021 Apr 3;38(8):3202–19. doi: 10.1093/molbev/msab092 (PMC8321546; doi:10.1093/molbev/msab092)
Supplement: msab092_Supplementary_Data [file msab092_supplementary_data.zip › SuppFig_combined_v2.pdf]

Figure S1

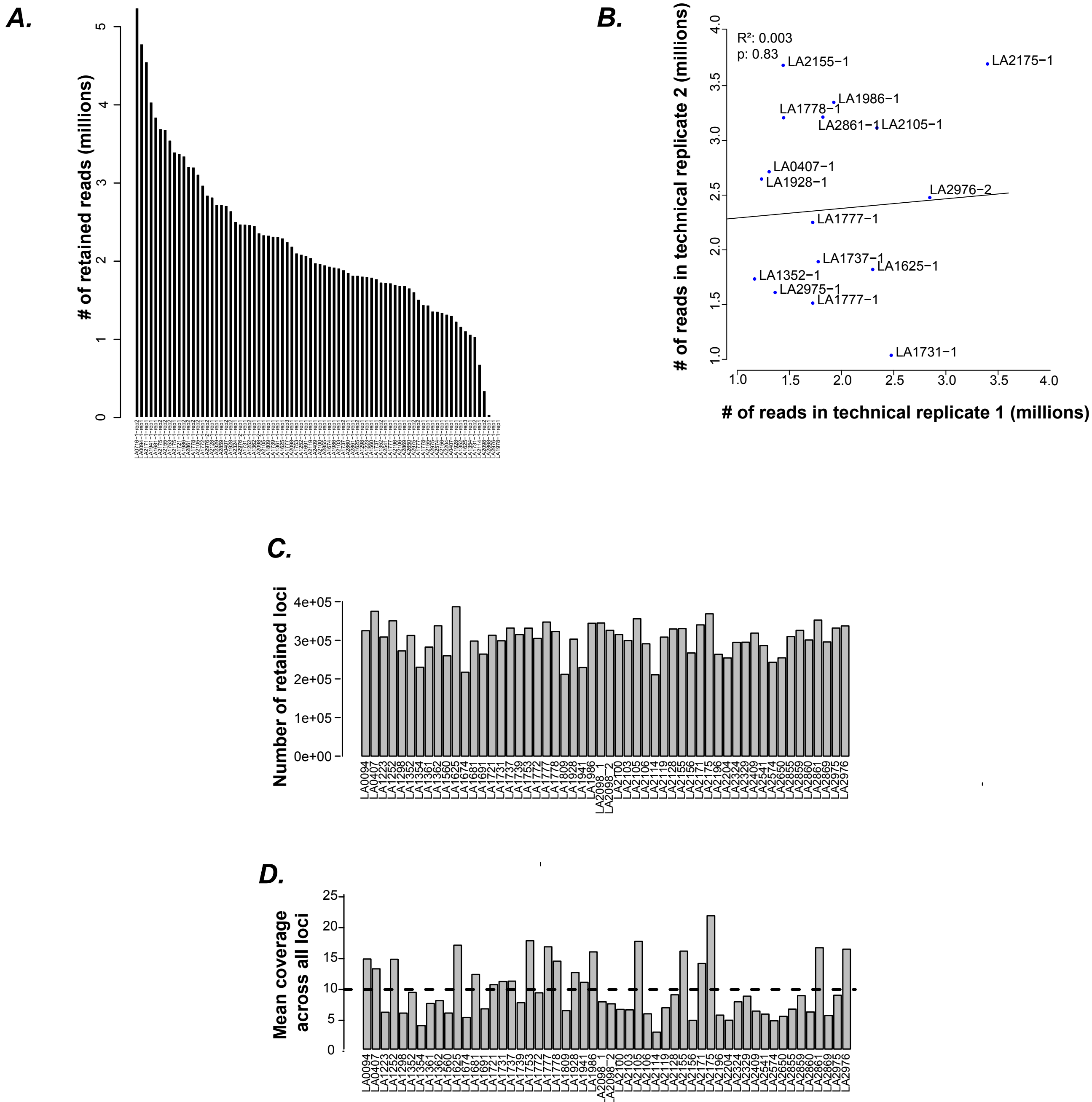

Supplementary Figure S1: Descriptive statistics of the RAD-seq data. (A) # of RAD-seq reads mapped to the LYC4 genome per accession (B) Difference in the number of reads between technical replicates observed is likely due to the randomness of restriction digestion and/or library preparation. -1 and -2 correspond to the first and second biological replicates of the accession. Most accessions had only one biological replicate. All technical replicate samples of the shown accessions were combined together for further analyses. (C) Number of retained loci and (D) Mean read coverage across all loci after running the Stacks pipeline, shown per accession.

Figure S2

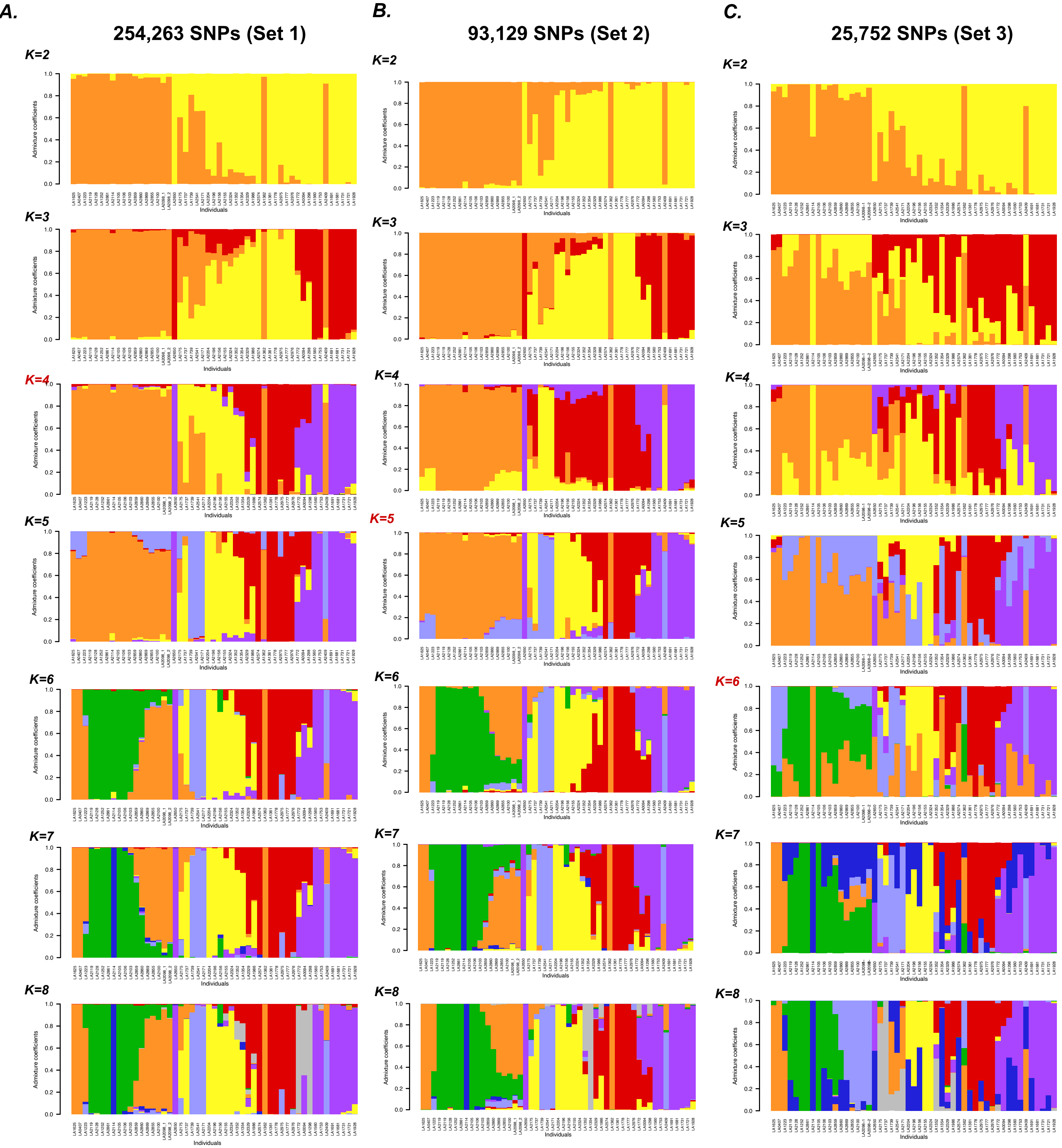

Supplementary Figure S2: Population structure plots obtained using LEA for (A) Set 1 (B) Set 2, (C) Set 3 marker sets. Best K, selected using the cross-entropy criterion, is highlighted in red. There was no substantial change in population assignment after K=6 in all three SNP sets. LA2114 was the only individual to be classified into a different population in K=7, however, the mean read coverage for this accession was the lowest (~2X) and the percentage of missing data the highest (29%, compared to <10% for most other accessions), leading to a lower confidence prediction of the seventh ancestral population.

Figure S3

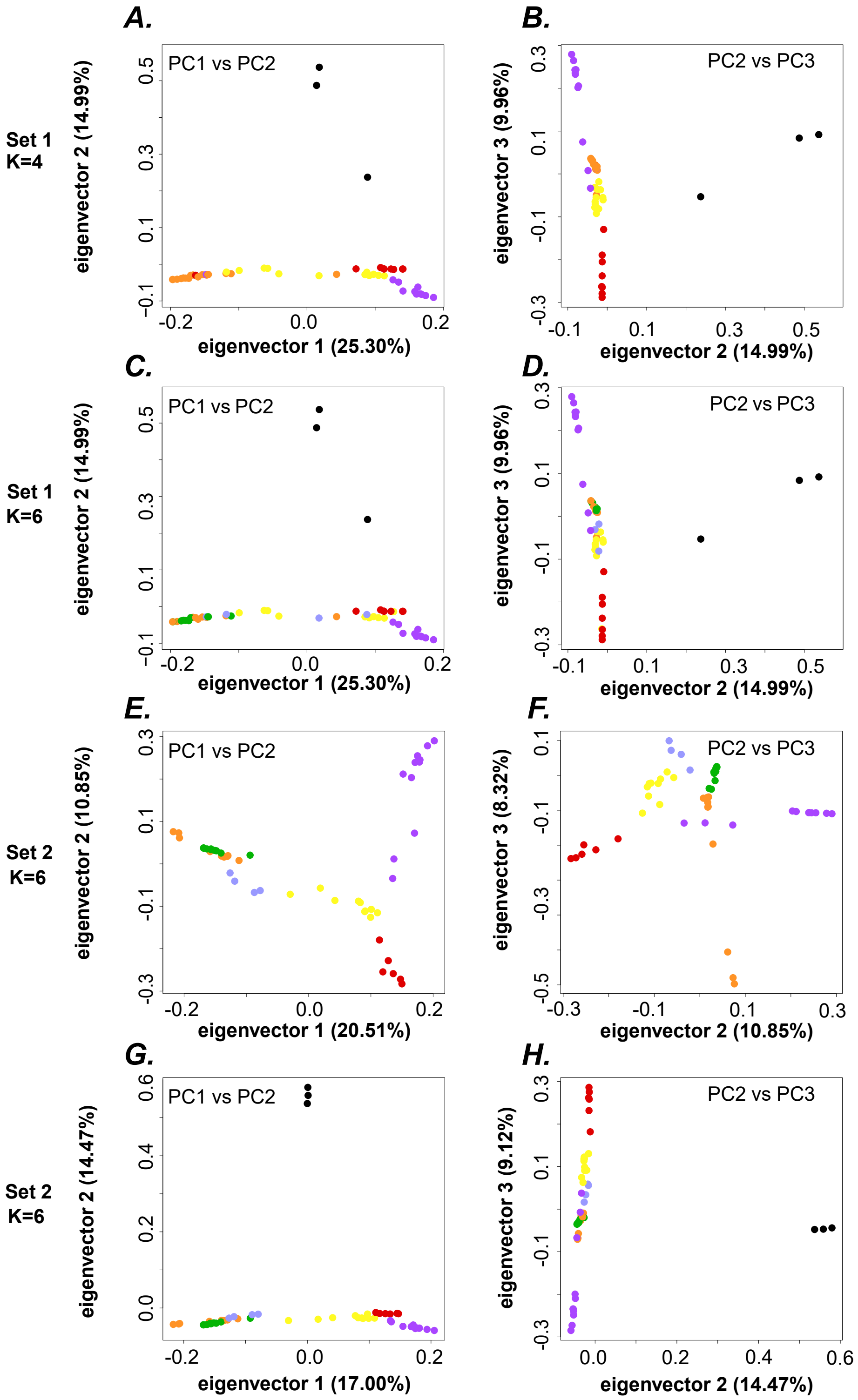

Supplementary Fig. S3: PCA plots showing population relatedness. Different factors tested here for clustering of accessions include # of population clusters (K's), variability explained by the first three PCs, SNP marker sets (Set 1,2), and presence/absence of *S. pennellii*

Figure S4

A.

Accession	# Bags with self fruit, each plant	# Fruits from hand self-pollinations	Pollen tube images (n)	Mating System
PI134417	3/4, 5/5	NT	3 accept, 0 reject (3)	SC
PI251305	1/3, 1/3	2/2, 3/3	NT	SC
PI390515	0/3,0/3	3/3, 2/2	2 accept, 0 reject (2)	SC
LA1252	3/3, 3/3	NT	NT	SC
LA2128	4/5, 2/2	NT	2 accept, 0 reject (2)	SC
LA2855	0/3, 0/4, 2/4	NT	1 accept, 6 reject (7)	MP
LA2860	1/1,1/1	NT	5 accept, 0 reject (5)	SC
LA4654	2/3, 5/7	NT	NT	SC
LA4655	3/3	NT	NT	SC
LA4656	3/3, 3/3	NT	NT	SC

B.

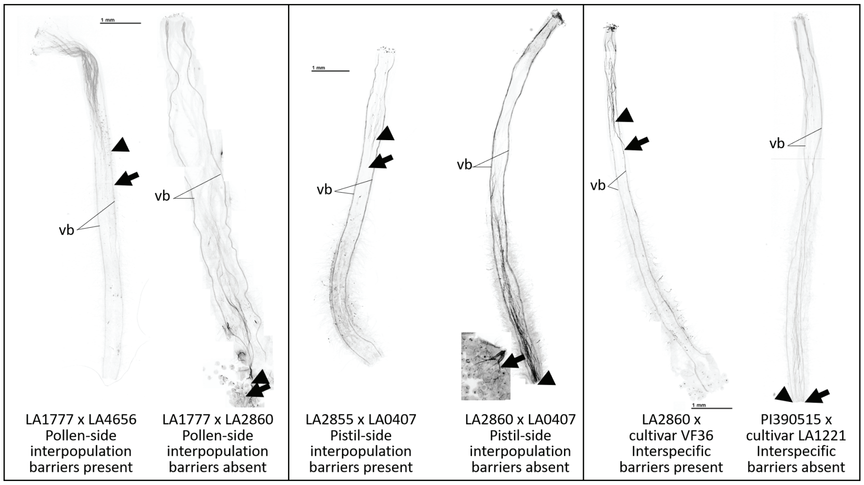

C.

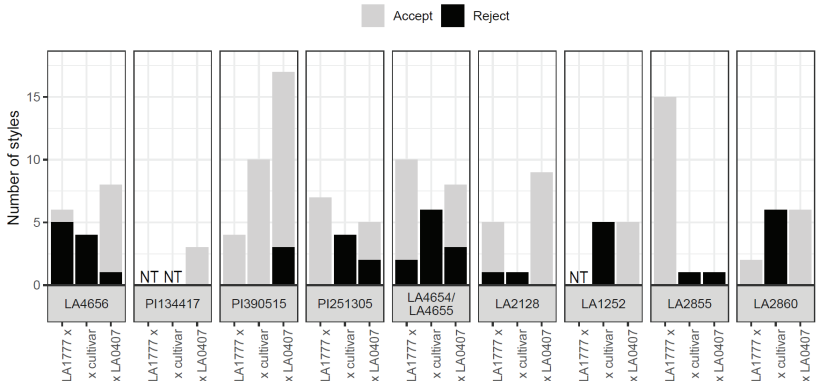

Supplementary Fig. S4: Definition of mating systems. (A) Determination of mating system for newly phenotyped accessions. Inflorescences were covered with net bags to exclude pollinators in order to evaluate whether fruits formed from passive self-pollination, and numbers in the second column indicate the number of bags containing self fruit for different individual. In some cases, hand pollinations were performed to ascertain whether self fruit could be formed, and numbers in the third column indicate the numbers of fruit formed for each hand-pollination for different individuals. In some cases, pollen tube growth in self pollinations was examined, and numbers in the fourth column indicate numbers of images showing pollen tube acceptance (>3 pollen tubes traverse the entire style) or rejection (<3 pollen tubes, typically none, traverse the entire style) with numbers in parentheses (n) indicating the number of individuals tested. The fifth column indicates the final assessment of mating system, SC = self-compatible, MP = mixed population with SC and SI (self-incompatible) individuals. NT = not tested.

(B) Examples of pollen tube growth analysis for detecting inter-population and interspecific barriers in newly phenotyped accessions. Images show pollen tubes in styles stained as described in Materials and Methods. The first pair of images shows crosses with *S. habrochaites* SI accession LA1777 as the pistil parent. If pollen tubes are rejected, the tested accession exhibits a pollen-side population barrier, here LA4656. If pollen tubes are accepted, this barrier is absent in the tested accession, here LA2860. The second pair of images shows crosses with *S. habrochaites* SC accession LA0407 as the pollen parent. If pollen tubes are rejected, the tested accession exhibits a pistil-side population barrier, here LA2855. If pollen tubes are not rejected, this barrier is absent in the tested accession, here LA2860. The third pair of images shows crosses with cultivated tomato (*S. lycopersicum*) as the pollen parent. If pollen tubes are rejected, the tested accession exhibits an interspecific barrier, known as unilateral incompatibility (UI), here LA2860. If pollen tubes are accepted, this barrier is absent in the tested accession, here PI390515. Arrowheads indicate the point at which most pollen tubes stop growing, arrows indicate the longest pollen tube, vb = vascular bundles, which are also stained.

(C) Histogram summarizing tests for inter-population and interspecific barriers in newly phenotyped accessions. Multiple tests were performed as described in Part B for each newly phenotyped accession used in this study. The number of style images showing pollen tube acceptance (>3 pollen tubes traverse the entire style), are shown in gray and images showing pollen tube rejection (<3 pollen tubes traverse the entire style) are shown in black. NT = not tested.

Figure S5

```
CLUSTAL multiple sequence alignment by MUSCLE (3.8)

                                                                                                            C1
LA2119_ORF                                   M  Y  K  S  Q  I  L  S  V  L  F  I  S  F  F  A  L  S  T  I  S  I  Y  G  S  F  D  H  W  Q  L  V  L  T  W
S.peruvianum_S13-RNase        ---------------------------------------------------CGTTTTTTGCTCTTTCAACCATATCCATTTATGGGTCATTCGACCATTGGCAACTTGTTTTGACTTGGC
LA2119_gDNA                   ---------------------AGTCACAGATCCTGTCAGTTCTCTTCATTTCGTTTTTTGCTCTTTCAACCATATCCATTTATGGGTCATTCGACCATTGGCAACTTGTTTTGACTTGGC
LA2119_StylarTranscript       CTTCAAACTAGAGAATGTATAAGTCACAGATCCTGTCAGTTCTCTTCATTTCGTTTTTTGCTCTTTCAACCATATCCATTTATGGGTCATTCGACCATTGGCAACTTGTTTTGACTTGGC
                                                                                 *********************************************************************

                                                               C2
LA2119_ORF                    P  A  G  F  C  K  T  K  D  C  P  R  K  D  I  P  N  N  F  T  I  H  G  V  W  P  D  H  T  S  F  V  M  Y  D  C  D  P  L  K
S.peruvianum_S13-RNase        CAGCAGGCTTCTGCAAAACTAAGGATTGTCCTAGAAAAGATATTCCAAACAACTTTACCATTCATGGCGTTTGGCCAGATCACACCTCCTTCGTCATGTATGACTGTGATCCCCTAAAAA
LA2119_gDNA                   CAGCAGGCTTCTGCAAAACTAAGGATTGTCCTAGAAAAGATATTCCAAACAACTTTACCATTCATGGCGTTTGGCCAGATCACACCTCCTTCGTCATGTATGACTGTGATCCCCTAAAAA
LA2119_StylarTranscript       CAGCAGGCTTCTGCAAAACTAAGGATTGTCCTAGAAAAGATATTCCAAACAACTTTACCATTCATGGCGTTTGGCCAGATCACACCTCCTTCGTCATGTATGACTGTGATCCCCTAAAAA
                              ************************************************************************************************************************

LA2119_ORF                    K  Y  K  T  I  D
S.peruvianum_S13-RNase        AGTATAAAACAATAGAT-------------------------------------------------------------------------------------------------------
LA2119_gDNA                   AGTATAAAACAATAGATGTAAGTGCAAACAGTCATTTTATCAAGCATCTTTTTATATATTTTTTTAAGTTCAACAATTTCCTTCTTGTTTGTTATTTATTTATTTAATCCACTCCTTGCC
LA2119_StylarTranscript       AGTATAAAACAATAGAT-------------------------------------------------------------------------------------------------------
                              *****************

LA2119_ORF                                   D  T  N  I  L  T  E  L  D  A  R  W  P  Q  L  T  S  T  K  I  I  G  L  Q  F  Q  R  F  W  E  Y  E  Y  R  K
S.peruvianum_S13-RNase        ---------------GATACAAATATACTCACTGAACTGGATGCCCGCTGGCCTCAATTGACTTCCACCAAAATAATTGGGTTGCAATTTCAAAGATTCTGGGAATATGAATATAGAAAG
LA2119_gDNA                   TATTGCCTACTACAGGATACAAATATACTCACTGAACTGGATGCCCGCTGGCCTCAATTGACTTCCACCAAAATAATTGGGTTGCAATTTCAAAGATTCTGGGAATATGAATATAGAAAG
LA2119_StylarTranscript       ---------------GATACAAATATACTCACTGAACTGGATGCCCGCTGGCCTCAATTGACTTCCACCAAAATAATTGGGTTGCAATTTCAAAGATTCTGGGAATATGAATATAGAAAG
                                             *********************************************************************************************************

                                 C3                                                       C4
LA2119_ORF                     H  G  T  C  C  A  D  V  F  N  Q  S  M  Y  F  D  I  S  M  K  L  T  D  S  I  D  L  L  K  I  L  R  T  K  G  I  K  P  G  Y
S.peruvianum_S13-RNase        CATGGAACGTGTTGTGCAGATGTCTTCAATCAATCTATGTATTTTGACATATCCATGAAATTAACAGATTCGATTGATCTTTTGAAAATTCTAAGAACAAAGGGAATTAAACCTGGATAT
LA2119_gDNA                   CATGGAACGTGTTGTGCAGATGTCTTCAATCAATCTATGTATTTTGACATATCCATGAAATTAACAGATTCGATTGATCTTTTGAAAATTCTAAGAACAAAGGGAATTAAACCTGGATAT
LA2119_StylarTranscript       CATGGAACGTGTTGTGCAGATGTCTTCAATCAATCTATGTATTTTGACATATCCATGAAATTAACAGATTCGATTGATCTTTTGAAAATTCTAAGAACAAAGGGAATTAAACCTGGATAT
                              ************************************************************************************************************************

                                                                                                                   C5
LA2119_ORF                     T  Y  T  G  D  Q  I  S  R  A  I  K  S  V  T  Q  N  N  P  N  P  K  C  T  Y  I  G  R  S  L  E  L  I  E  I  G  I  C  F  N
S.peruvianum_S13-RNase        ACTTATACCGGTGACCAAATCAGTCGTGCCATTAAGTCAGTTACTCAAAATAATCCAAACCCCAAGTGCACTTATATAGGGAGAAGCTTGGAACTAATTGAGATAGGCATATGTTTCAAC
LA2119_gDNA                   ACTTATACCGGTGACCAAATCAGTCGTGCCATTAAGTCAGTTACTCAAAATAATCCAAACCCCAAGTGCACTTATATAGGGAGAAGCTTGGAACTAATTGAGATAGGCATATGTTTCAAC
LA2119_StylarTranscript       ACTTATACCGGTGACCAAATCAGTCGTGCCATTAAGTCAGTTACTCAAAATAATCCAAACCCCAAGTGCACTTATATAGGGAGAAGCTTGGAACTAATTGAGATAGGCATATGTTTCAAC
                              ************************************************************************************************************************

LA2119_ORF                     R  T  T  N  A  L  M  P  C  P  R  I  S  T  S  C  K  L  G  T  V  E  G  V  K  F  R  *
S.peruvianum_S13-RNase        CGAACAACAAATGCTCTGATGCCATGCCCTCGAATCAGTACGTCATGCAAGTTAGGGACCTTAGAAGGGGTAAAGTTTCGATGATCATCTCTTTCTTTCTTTTTCTTGATAATGTATAAT
LA2119_gDNA                   CGAACAACAAATGCTCTGATGCCATGCCCTCGAATCAGTACGTCATGCAAGTTAGGGACCGTAGAAGGGGTAAAGTTTCGATGATCATCTCTTTCTTTCTTTTTCTTGATAATGTATAAT
LA2119_StylarTranscript       CGAACAACAAATGCTCTGATGCCATGCCCTCGAATCAGTACGTCATGCAAGTTAGGGACCGTAGAAGGGGTAAAGTTTCGATGATCATCTCTTTCTTTCTTTTTCTTGATAATGTATAAT
                              ************************************************************ ***********************************************************

S.peruvianum_S13-RNase        AAAACGTCAGAGACATCCGTGACCGACATAACTATAAAGAAAAATGTTATTCTTGTTGACGGCATTACGGAACCAGTGGAAGGGAATGTCTTGTTAATCAATTTATGATCAGTACAAAAT
LA2119_gDNA                   AAAACG-CAAAGACATCCGTGACCGACATAACTATAAAGAAAAA---------------------------------------------------------------------------
LA2119_StylarTranscript       AAAACG-CAAAGACATCCGTGACCGACATAACTATAAAGAAAAATGTTATTCTTGTTGACGGCATTACGGAACCAGTGGAAGGGAATGTCTTGTTAATCAATTTATGATCAGTACAAAAT

                              ****** ** **********************************

S.peruvianum_S13-RNase        AGCCATACTCAAGATATGAATGTTGGATACCTCGTT
LA2119_gDNA                   ------------------------------------
LA2119_StylarTranscript       AGCCATACTCAAGATATGAATGTTGG----------
```

Supplementary Fig. S5: The hab-7 S-RNase allele from SC-1 S. habrochaites accession LA2119. Alignment of *hab-7 S-RNase* cDNA and genomic DNA coding sequences is shown (in red), with the intron shown in black. The top line shows the encoded amino acid sequence (ORF). For comparison, alignment with *S. peruvianum* S13 S-RNase sequences is shown. Conserved *S-RNase* regions C1-C5 are highlighted.

Figure S6

```
HT-A genomic sequence alignment  CLUSTAL multiple sequence alignment by MUSCLE (3.8
                                                          )

               M  A  F  K  A  N  I  L  L  I  F  S  L  V  F  M  V  I  S  S  E  V  I  A  R  E  M  V  E
LA0407*       ATGGCATTCAAGGCAAATATTTTGCTTATATTTTCTTTGGTTTTTATGGTTATATCATCAGAGGTTATTGCAAGGGAAATGGTTGAGGGTAAGTTGTTCTAATTGTAGTTTTAAGTACTA
LA4655-1      -----------------TATTTTGCTTATATTTTCTTTGGTTTTTATGGTTATATCATCAGAGGTTATTGCAAGGGAAATGGTTGAGGGTAAGTTGTTCTAATTGTAGTTTTAAGTACTA
LA4655-2      ----------------ATATTTTGCTTATATTTTCTTTGGTTTTTATGGTTATATCATCAGAGGTTATTGCAAGGGAAATGGTTGAGGGTAAGTTGTTCTAATTGTAGTTTTAAGTACTA
LA1223        ATGGCATTCAAGGCAAATATTTTGCTTATATTTTCTTTGGTTTTTATGGTTATATCATCAGAGGTTATTGCAAGGGAAATGGTTGAGGGTAAGTGGTTCTAATTGTAGTTTTAAGTACTA
PI1390515     ATGGCNTTCAAGGCAAATATTTTGCTTATATTTTCTTTGGTTTTTATGGTTATATCATCAGAGGTTATTGCAAGGGAAATGGTTGAGGGTAAGTGGTTCTAATTGTAGTTTTAAGTACTA
PI251305      ATGNCATTCAAGGCAAATATTTTGCTTATATTTTCTTTGGTTTTTATGGTTATATCATCAGAGGTTATTGCAAGGGAAATGGTTGAGGGTAAGTGGTTCTAATTGTAGTTTTAAGTACTA
LA1777*       ATGGCATTCAAGGCAAATATTTTGCTTATATTTTCTTTGGTTTTTATGGTTATATCATCAGAGGTTATTGCAAGGGAAATGGTTGAGGGTAAGTTGTTCTAATTGAAGTTTTAAGTACTA
                               ***************************************************************************** ********** **************

LA0407*       ATTACAAATTTCATATGACAAATTAAATTAAGTGCATAATGGGATATGGTCAAAGAGAACTTTTACTTTATGAAATATCTTAGTTTTACTCTTTTGTTTTATAGCCTTGTTATTAGCAAA
LA4655-1      ATTACAAATTTCATATGACAAATTAAATTAAGTGCATAATGGGATATGGTCAAAGAGAACTTTTACTTTATGAAATATCTTAGTTTTAC-CCTTTGTTTTATAGCCTTGTTATTAGCAAA
LA4655-2      ATTACAAATTTCATATGACAAATTAAATTAAGTGCATAATGGGATATGGTCAAAGAGAACTTTTACTTTATGAAATATCTTAGTTTTACTCTTTTGTTTTATAGCCTTGTTATTAGCAAA
LA1223        ATTACAAATTTCATATGACAAATTAAATTAAGTGCATAATGGGATATGGTCAAAGAGAACTTTTACTTTATGAAATATCTTAGTTTTAC-CCTTTGTTTTATAGCCTTGTTATTAGCAAA
PI1390515     ATTACAAATTTCATATGACAAATTAAATTAAGTGCATAATGGGATATGGTCAAAGAGAACTTTTACTTTATGAAATATCTTAGTTTTAC-CCTTTGTTTTATAGCCTTGTTATTAGCAAA
PI251305      ATTACAAATTTCATATGACAAATTAAATTAAGTGCATAATGGGATATGGTCAAAGAGAACTTTTACTTTATGAAATATCTTAGTTTTAC-CCTTTGTTTTATAGCCTTGTTATTAGCAAA
LA1777*       ATTACAAATTTCATATCACAAATTAAATTAAGTGCATAATGGCATATGGTCAAAGAGAACTTTTACTTTATGAAATATCTTAGTTTTAC-CCTTTGTTTTATAGCCTTGTTATTAGCAAA
              **************** ************************* ********************************************** * ****************************

LA0407*       ------TCGTCTCATGCAAATGAGTAATTTTGAACCTTTTTTTTTACAAAAGTATTGTAATACGTAAAACTCAACGTCTTTATTGGAGCTCATTTATATTTTAAAATTAAGAGCAAATAT
LA4655-1      ------CCGTCTCATGCAAATGAGTAATTTTGAACCTTTTTTTTACA-AAAGTATTGTAATACGTAAAACTCAACGTCTATATTGAAGCTCATTTAAATTTTAAAATTAAGCGCAAATAT
LA4655-2      ------TCGTCTCATGCAAATGAGTAATTTTGAACCTTTTTTTTTACAAAAGTATTGTAATACGTAAAACTCAACGTCTTTATTGGAGCTCATTTATATTTTAAAATTAAGAGCAAATAT
LA1223        ------TCGTTTCATGCAAATGAGTAATTTTGAACCTTTTTTTTTTACAAAGTATTGTAATATGTAAAACTCAACGTCTATATTGAAGCTCATTTAAATTTTAAAATTAAGCGCAAATAT
PI1390515     ------TCGTTTCATGCAAATGAGTAATTTTGAACCTTTTTTTTTTACAAAGTATTGTAATATGTAAAACTCAACGTCTATATTGAAGCTCATTTAAATTTTAAAATTAAGCGCAAATAT
PI251305      ------TCGTTTCATGCAAATGAGTAATTTTGAACCTTTTTTTTTTACAAAGTATTGTAATATGTAAAACTCAACGTCTATATTGAAGCTCATTTAAATTTTAAAATTAAGCGCAAATAT
LA1777*       TCGTCTTCGTCTCATGCAAATGAGTAATTTTGAAC--TTTTTTTTTACAAAGTATTGTAATACTTAAAACTCAACGTCTATATTGGAGCTCATTTATATTTTAAAATTAAGAGCAAATAT
                     *** ************************  *******    **************  *************** ***** ********** ************** ********

LA0407*       GAGAGAATTTACTAGTGTAAAACTATATAAAATAGACCCAAAATATAATTAAGGGTAAAATTAAGAAATCTTATATAGTAGCATTAGTTGACTTGTCTAAGCAACCATTGATCCCACATG
LA4655-1      GAGAGAATTTACTAGTGTAAAACTATATAAAATAGA-CCAAAATATAATTAAGGGTAAAATTAAGAAATCTTATATAGTAGCATTAGTTGACTTGTCTAAGCAACCATTGATCCCACATG
LA4655-2      GAGAGAATTTACTAGTGTAAAACTATATAAAATAGACCCAAAATATAATTAAGGGTAAAATTAAGAAATCTTATATAGTAGCATTAGTTGACTTGTCTAAGCAACCATTGATCCCACATG
LA1223        GAGAGAATTTACTAGTGTAAAACTATATAAAATAGA-CCAAAATATAATTAAGGGTAAAATTAAGAAATCTTATATAGTAGCATTAGTTGACTTGTCTAAGCAACCATTGATCCCACATG
PI1390515     GAGAGAATTTACTAGTGTAAAACTATATAAAATAGA-CCAAAATATAATTAAGGGTAAAATTAAGAAATCTTATATAGTAGCATTAGTTGACTTGTCTAAGCAACCATTGATCCCACATG
PI251305      GAGAGAATTTACTAGTGTAAAACTATATAAAATAGA-CCAAAATATAATTAAGGGTAAAATTAAGAAATCTTATATAGTAGCATTAGTTGACTTGTCTAAGCAACCATTGATCCCACATG
LA1777*       GAGAGAATTTACCAGTGTAAAACTATATAAAATAGACCCAAAATATAATTAAGGGTAAAATTAAGAAATCTTATATAGTAGCATTAGTTGACTTGTCTAAGCAACCATTGATCCCACATG
              ************ *********************** ***********************************************************************************

LA0407*       AAAAAGGTGGTATATTTGACTATATAGAG-AAAAAAAGAAAATTATAATAATTACTATTAAAATTAACATGGAAGAATTAAGAAAATGTTATGAACTTCATTATTATAATTTGATTTTTG
LA4655-1      AAAAAGGTGGTATATTTGACTATATAGAG-AAAAAAAGAAAATTATAATAATTACT--------------------ATGAAGAAAATGTTATGAACTTCATTATTATATTTTGATTTTTG
LA4655-2      AAAAAGGTGGTATATTTGACTATATAGAG-AAAAAAAGAAAATTATAATAATTACTATTAAAATTAACATGGAAGAATTAAGAAAATGTTATGAACTTCATTATTATAATTTGATTTTTG
LA1223        AAAAAGGTGGTATATTTGACTATATAGAGAAAAAAAAGAAAATTATAATAATTACT--------------------ATGAAGAAAATGTTATGAACTTCATTATTATATTTTGATTTTTG
PI1390515     AAAAAGGTGGTATATTTGACTATATAGAGAAAAAAAAGAAAATTATAATAATTACT--------------------ATGAAGAAAATGTTATGAACTTCATTATTATATTTTGATTTTTG
PI251305      AAAAAGGTGGTATATTTGACTATATAGAGAAAAAAAAGAAAATTATAATAATTACT--------------------ATGAAGAAAATGTTATGAACTTCATTATTATATTTTGATTTTTG
LA1777*       AAAAAGGTGGTATATTTGACTATATAGAGAAAAAAAAGAAAATTATAATAATTACT--------------------ATGAAGAAAATGTTATGAACTTCATTATTATATTTTGATTTTTG
              ***************************** **************************                    ** ***************************** ***********

                                                                                  A  N  Q  V  Q  N  S  F  E  L  N  N  P  T  L  Q  K  K  G
LA0407*       AACATATTTCATTTTTTCTGTTCTCTAACAAAATTATTTGTACACAT---CAATTTTGGTGCAGCAAATCAAGTTCAAAATTCATTTGAATTGAATAATCCGACACTTCAGAAAAAAGGT
LA4655-1      AACATATTTCATTTTTTCTGTTCTCTAACAAAATTATTTGTACACATGACCAATTTTGGTGCAGCAAATCAAGTTCAAAATACATTTGAATTGAATAATCCGACACTTCAGAAAAAAGGT
LA4655-2      AACATATTTCATTTTTTCTGTTCTCTAACAAAATTATTTGTACACAT---CAATTTTGGTGCAGCAAATCAAGTTCAAAATTCATTTGAATTGAATAATCCGACACTTCAGAAAAAAGGT
LA1223        AACATATTTCATTTTTTCTGTTCTCTAACAAAATTATTTGTACACATGATCAATTTTGGTGCAGCAAATCAAGTTCAAAATACATTTGAATTGAATAATCCGACACTTCAAAAAAAAGGT
PI1390515     AACATATTTCATTTTTTCTGTTCTCTAACAAAATTATTTGTACACATGATCAATTTTGGTGCAGCAAATCAAGTTCAAAATACATTTGAATTGAATAATCCGACACTTCAGAAAAAAGGT
PI251305      AACATATTTCATTTTTTCTGTTCTCTAACAAAATTATTTGTACACATGATCAATTTTGGTGCAGCAAATCAAGTTCAAAATACATTTGAATTGAATAATCCGACACTTCAGAAAAAAGGT
LA1777*       AACATATTTCATTTTTTCTGTTCTCTAACAAAATTATTTGTACACAT---CAATTTTGGTGCAGAAAATCAAGTTCAAAATACATTTGAATTGAATAATCCGACACTTCAGAAAAAAGGT
              ***********************************************   ************** **************** **************************** *********

               G  G  S  L  F  P  N  I  A  C  L  G  C  S  C  P  K  K  D  N  K  N  N  N  N  N  N  N  N  N  D  D  D  D  D  D  D  S  F  I
LA0407*       GGGGGATCATTATTTCCTAATATAGCGTGTTTGGGTTGCAGTTGCCCAAAAAAAGATAATAAAAACAATAATAATAATAATAATAATAACGATGACGATGATGACGATGATAGTTTCATT
LA4655-1      GGGGGATCATTATTTCCTAATATAGCGTGTTTGGGTTGCAGTTGCCCAAAAAAAGATAATAAAAACAATAATAATAATAATAATAATAACGATGACGATGATGACGATGATAG-------
LA4655-2      GGGGGATCATTATTTCCTAATATAGCGTGTTTGGGTTGCAGTTGCCCAAAAAAAGATAATAAAAACAATAATAATAATAATAATAATAACGATGACGATGATGACGATGATAG-------
LA1223        GGGGGATCATTATTTCCTAATATAGCGTGTTTGGGTTGCAGTTGCCCAAAATAAGATAATAAAAAC------AATAATAATAATAATAACGATGACGATGATGACGATGACAGTTTCATT
PI1390515     GGGGGATCATTATTTCCTAATATAGCGTGTTTGGGTTGCAGTTGCCCAAAATAAGATAATAAAAAC------AATAATAATAATAATAACGATGACGATGATGACGATGACAG-------
PI251305      GGGGGATCATTATTTCCTAATATAGCGTGTTTGGGTTGCAGTTGCCCAAAATAAGATAATAAAAAC------AATAATAATAATAATAACGATGACGATGATGACGATGA----------
LA1777*       GGGGGATCATTATTTCCTAATATAGCGTGTTTGGGTTGCAGTTGCCCAAAAAAAGATAATAAAAAC------AATAATAATAATAATAACGATGACGATGATGACGATGACAGTTTCATT
              *************************************************** **************      **************************************

               G  N  V  C  K  A  M  C  C  *
LA0407*       GGTAATGTTTGTAAAGCCATGTGTTGTTAG
LA4655-1      ------------------------------
LA4655-2      ------------------------------
LA1223        GGTAATGTTTGTAAAGCCATGTGTTGTTAG
PI1390515     ------------------------------
PI251305      ------------------------------
LA1777*       GGTAATGTTTGTAAAGCCATGTGTTGTTAG
```

Supplementary Fig. S6: Alignment of HT-A genomic DNA sequences. HT-A sequences were amplified using gene specific primers and PCR products (LA4655, PI1251305, PI1390515) or clones of PCR products (LA1223) were subject to Sanger sequencing. Sequences were aligned with references from LA0407 (Genbank GU362659.1) and LA1777 (GU362649.1). Exons are displayed in red, and the deduced amino acid sequence is denoted above the exons. The A->T SNP leading to a nonsense mutation (K->stop) in exon 2 that was discovered in some northern accessions is highlighted in gray. *Covey et al. 2010

Figure S7

A. **B.**

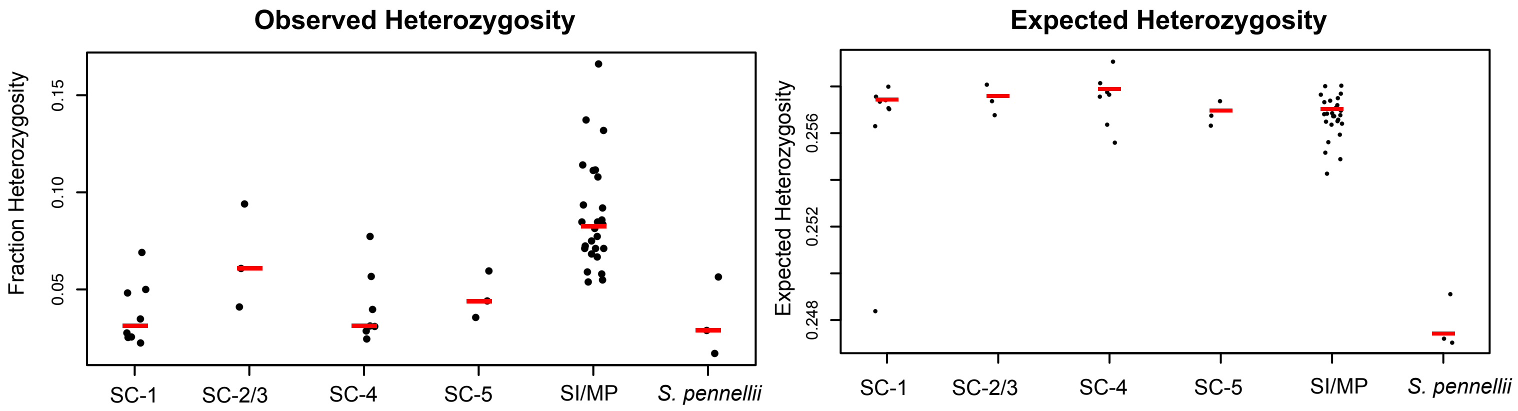

SC-1	
LA2128	0.07
LA2119	0.05
LA2114	0.05
LA1252	0.03
LA2106	0.03
LA2103	0.03
LA2105	0.03
LA2861	0.02

SC-2/SC-3	
LA0407	0.09
LA1223	0.06
LA1625	0.04

SC-4	
LA1721	0.08
LA1753	0.06
LA1731	0.04
LA1560	0.03
LA1928	0.03
LA1691	0.03
LA1681	0.02

SC-5	
LA2869	0.06
LA2860	0.04
LA2859	0.04

SI/MP	
LA2098_1	0.17
LA2156	0.14
LA1737	0.13
LA2175	0.11
LA1777	0.11
LA1986	0.11
LA2976	0.11
LA1352	0.09
LA1739	0.09
LA2098_2	0.09
LA2541	0.08
LA1298	0.08
LA2324	0.08
LA1778	0.08
LA0094	0.08
LA1354	0.08
LA1772	0.07
LA2100	0.07
LA2204	0.07
LA2196	0.07
LA2155	0.07
LA2171	0.07
LA1361	0.06
LA2329	0.06
LA2855	0.05
LA2574	0.05

S. pennellii	
LA1674	0.06
LA1809	0.03
LA1941	0.02

C.

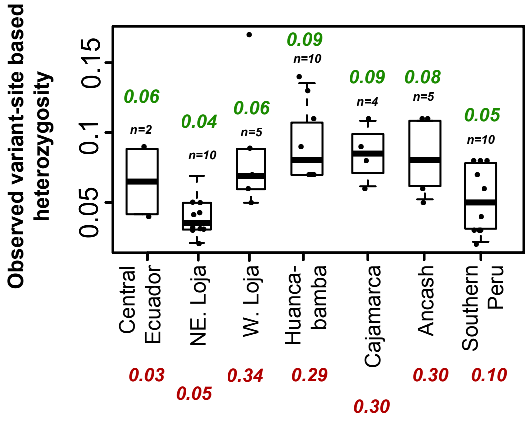

Supplementary Fig. S7: Heterozygosity estimates **(A,B)** Observed and expected heterozygosity obtained using variant sites divided based on the SC groups as described in the main text. Median value is shown using the red line. **(C)** Variant sites-derived estimates of heterozygosity for each ecogeographic group using vcfTools.

Figure S8

A.

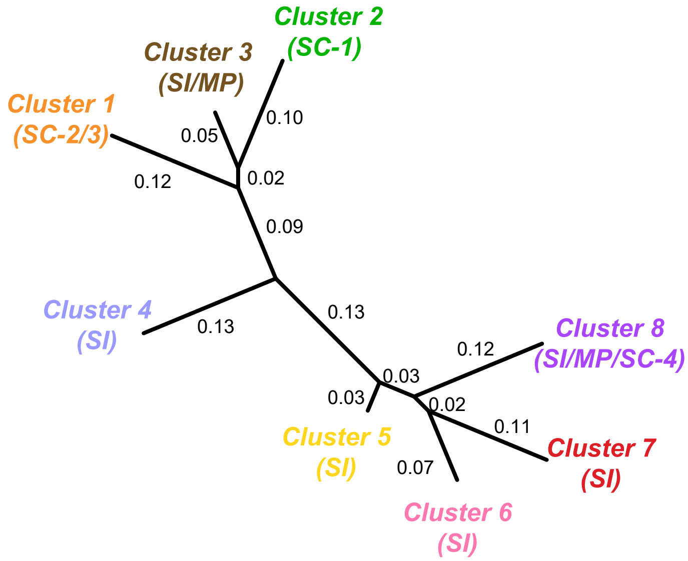

B.

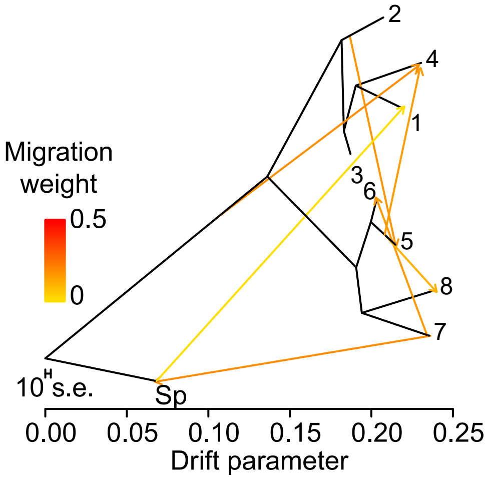

Allowed Migration Events	Log likelihood	LRT (test statistic, p-value)	% variance explained
0	-589.27	--	0.991034
1	57.81	1294.15 (2.11e-283)	0.997558
2	114.99	114.37 (1.08e-26)	0.998156
3	241.72	252.46 (4.57e-57)	0.9992747
4	265.97	48.49 (3.32e-12)	0.9994937
5	285.60	39.28 (3.68e-10)	0.999637
6	302.82	34.43 (4.42e-09)	0.9998275
7	311.70	17.78 (2.49e-05)	0.9999293
8	309.87	-3.76 (1)	0.9999032
9	314.70		0.9999473
10	319.57		0.9999369

Supplementary Fig. S8: Fixation Index and TreeMix results. **(A)** Neighbor joining tree of Fst values. Leaf nodes are the cluster IDs and branch lengths correspond to Fst values. **(B)** TreeMix analysis showing inferred migration events between different clusters. Migration weight indicates confidence in a given inferred migration event. Tree was obtained using maximum likelihood analysis by the TreeMix software. Between 0 and 8 migration events were modeled onto the ML tree. Statistics associated with each migration event are described in the table. The LR test statistic and p-values refer to the likelihood of observing a given number of migration events (as noted in the first column) compared to the number of migration events in the first column of the previous row.

Figure S9

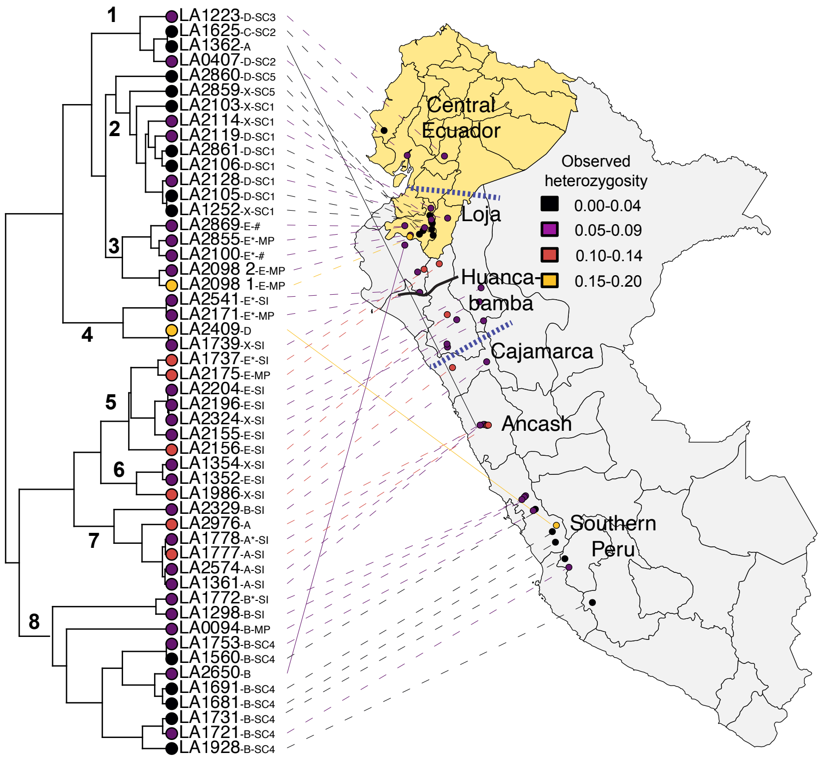

Supplementary Figure S9: Summary of phenotypes measured for S. habrochaites. Heterozygosity estimates from variant sites are associated with the phylogenetic tree obtained using coalescent analysis. Yellow highlighted regions corresponds to Ecuador, while the rest of the map represents Peru. The AHZ spans the region bounded by the blue lines, with the Huancabamba Depression indicated by the solid black curve. Named regions are as per Sifres et al, 2011. Letters after accession numbers stand for acylsugar chemotype based on Kim et al, 2012 and Fig. 5 (if marked by *), followed by SC/SI/MP assignment as per Table 1. X=no chemotype assignment, #=mating system not assessed.
