# Supplementary material for: Migration through a Major Andean Ecogeographic Disruption as a Driver of Genetic and Phenotypic Diversity in a Wild Tomato Species
Source: Mol Biol Evol. 2021 Apr 3;38(8):3202–19. doi: 10.1093/molbev/msab092 (PMC8321546; doi:10.1093/molbev/msab092)
Supplement: msab092_Supplementary_Data [file msab092_supplementary_data.zip › SupplementaryText_1_v5.pdf]

Supplementary Text 1

A.1 Impact of TGRC stock regeneration on data analysis

S. habrochaites accessions have been collected over the last 70 years, with the earliest accession LA0094 collected in 1948, and most accessions collected in the 1970s. One concern with using such old accessions is creation of a genetic bottleneck that can affect heterozygosity estimates and population relatedness. However, TGRC takes care to preserve the original genetic diversity of individual accessions, including (i) collecting seeds from multiple plants to mimic native variability, (ii) maintaining sufficiently large populations (i.e. >50 plants for outcrossing species), (iii) maintaining seed stocks at low temperatures and humidity, (iv) performing mass sib crosses from bulk pollen, (v) reducing intervals between seed generation, and (vi) testing germination efficiencies every 2-3 years after the first 10 years. Most accessions are regenerated from seed once every 20 years or more. The earliest *S. habrochaites* accession collected was in 1948 (LA0094; ~3-4 regenerations), while most accessions were collected in the 1970s and 1980s (~2-3 regenerations). Given all these efforts to maintain the genetic diversity, we believe that the effect on heterozygosity estimations and population relationships will be minimal. Nonetheless, if there are any effects, they would contribute to reduced heterozygosity within populations, particularly for SI populations with small original collections. In contrast, the strictly autogamous/selfing populations may experience an increase in heterozygosity. Due to lack of specific information on several of these steps, it is difficult to model their effects into Structure analysis. However, as noted above, we believe the effects would be minimal due to low number of regenerations and maintenance of genetic diversity at TGRC.

A.2 Impact of RAD-seq coverage on heterozygosity analysis

The primary concern with low coverage is the inability to differentiate heterozygous loci from sequencing errors. In our sample runs, the average sequencing error rate across all 100 sequenced bases was 0.04% **(Supplementary Text Fig. 1),** much lower than the default upper bound error rate of Stacks v1.44 (10%), which can lead to overestimation of homozygosity. Thus, in our previous analysis with Stacks v1.44 using Set 3 SNPs, we had used a much reduced higher bound of 2%, which would substantially minimize wrong Het calls.

Stacks v2.3, whose results are primarily used for the major inferences in the manuscript, implements a Bayesian Genotype Caller (BGC), and users are unable to set specific error rates. BGC, in addition to sequencer base calls, also makes use of data from other individuals for inferring allelic states of loci whose states are not robustly predicted. Prior simulation studies have shown that BGC accurately estimates allelic states from low-coverage RAD-seq data (Maruki and

Lynch 2017). This paper refers to average 7X coverage as low, 10X coverage as moderately high, and 17X coverage as very high. Our median coverage was ~9.5X - at this level, all approaches produce correct allelic base calls (Maruki and Lynch 2017). The performance of BGC is also highest in low-coverage data from humans, which are obligately allogamous. Due to these reasons, we believe our H, Fst estimates and population relationships are robust to effects of low-coverage.

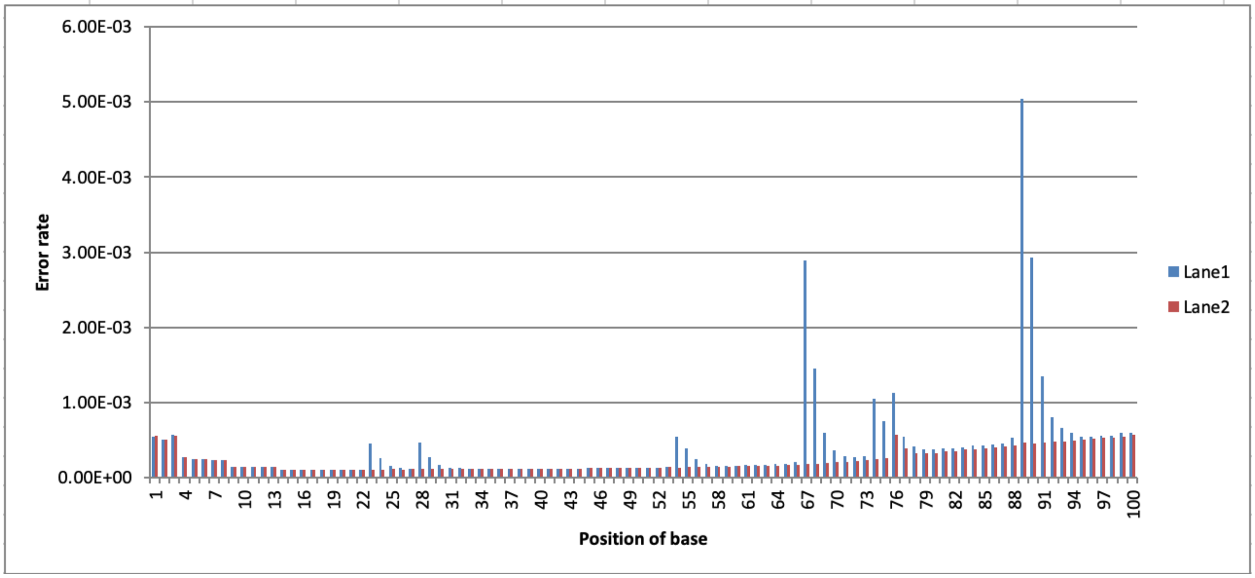

Supplementary Text Fig. 1: Error rates of Illumina sequencing runs obtained from the sequencing center.

Our initial filtering parameters to retain only high-quality SNPs were with the following parameters in vcftools *(--max-missing 0.8 --min-meanDP 6 --max-meanDP 30 --maf 0.05 --mac 3*) which retained 93,129 SNPs. To explore the effects that read depth had on our ability to determine heterozygous individuals/sites, we calculated heterozygosity values and average read depth in vcftools with different filtering parameters for read depth.

Supplementary Text Table 1: Effect of read depth filtering on number of SNPs left

Minimum mean read depth threshold	# of SNPs left	Average read depth per accession
6x [original]	93,129	9.5x
10x	24,855	12.9x
15x	2,589	18.5x

Minimum read depth threshold	# of SNPs left	Average read depth per accession
4x	29,263	11x
6x	3,550	13.2x
8x	137	15.1x

While the absolute values of observed heterozygosity do change with the different parameters of filtering, the general patterns are quite robust. When plotting the original values of heterozygosity to the values calculated using a stricter filtering of minimum mean depth of 10x, the best fit line has an R^2 value of 0.9792 **(Supplementary Text Fig. 2)**.

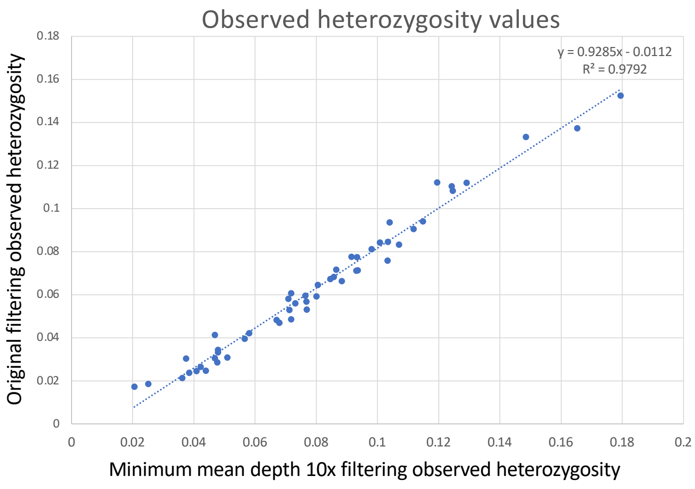

Supplementary Text Fig. 2: Observed heterozygosity values calculated with vcftools for the original filtering of SNPs and more strict filtering of a minimum mean depth of 10x for a given locus.

A.3 Using a variant-free approach for making Het calls

Calculating observed heterozygosity from only sites included in a VCF may give biased estimates since invariant sites are ignored. Therefore, we calculated heterozygosity from the BAM files after mapping reads of each accession to the selected reference genome using ANGSD (Korneliussen et al. 2014). The resulting values of observed heterozygosity range from 0.0006-0.015 for the ANGSD approach and 0.017-0.154 for vcftools. Even though the absolute values between the two methods were quite different, most of the samples show a strong correlation. With all of the samples included the best fit line only has an R^2 value of 0.56 **(Supplemental Text**

Fig. 3). However, three accessions (LA1941, LA1809, and LA1674) show large variation between the two methods. If those three outliers are removed, then the R2 value increases to 0.94 **(Supplemental Text Fig. 4)**.

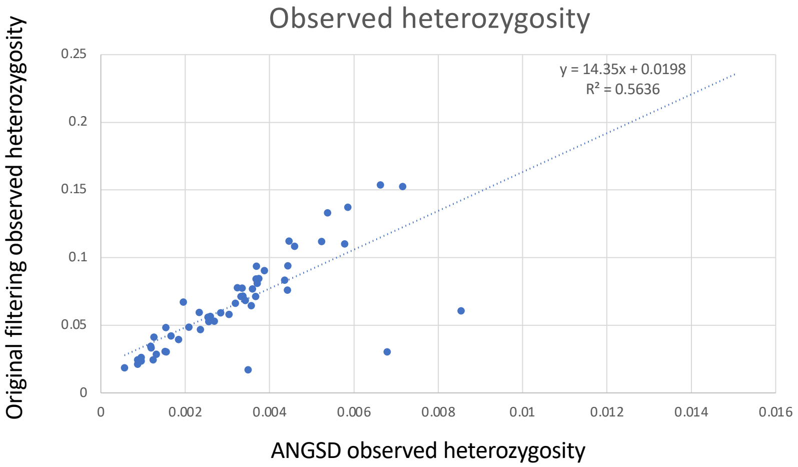

Supplemental Text Fig. 3. Observed heterozygosity calculated by vcftools and ANGSD. All samples are included.

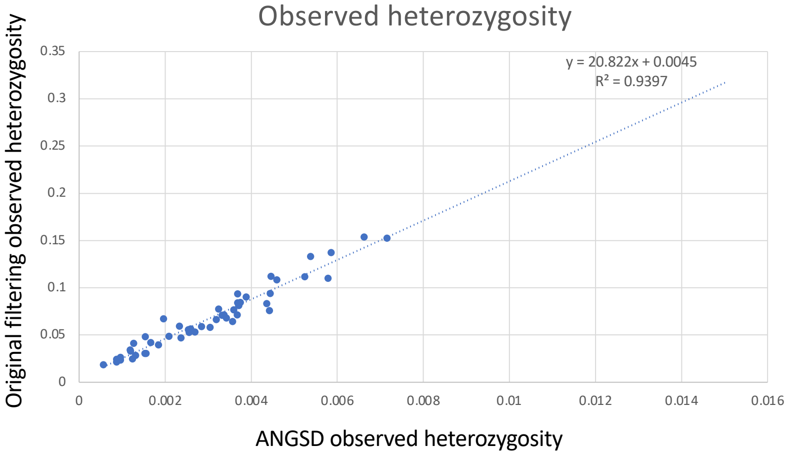

Supplemental Text Fig. 4. Observed heterozygosity calculated by vcftools and ANGSD with the three outlier samples (LA1941, LA1809, and LA1674) removed.

A.4 Impact of minor allele frequency thresholds on population structure

As shown previously by Linck and Battey (2019) different thresholds for minor allele frequency (MAF) can have drastic impacts on the number of SNPs retained and inferred

population structure (Linck and Battey 2019). We explored the robustness and choice of our filtering parameters by testing a range of MAF values from 0 (no filtering) to a MAF value of 0.1. The number of SNPs retained varied from 342,884 with no filtering to 74,755, with no other filtering put in place. A PCA was conducted on the resulting VCF files for MAF filtering of 0, 0.02, 0.04, 0.06, 0.08, and 0.1 using the same commands for SNPRelate (see Methods). The resulting plots of PC1 vs PC2 show a very similar pattern. We do see the relationships with no MAF filtering were obscure and we did not recover the populations as in other data sets. This is not surprising though since Linck and Battey 2019 make a point that the noise from these additional singletons may be masking the "true" signal from the informative alleles which has been observed in PCA.

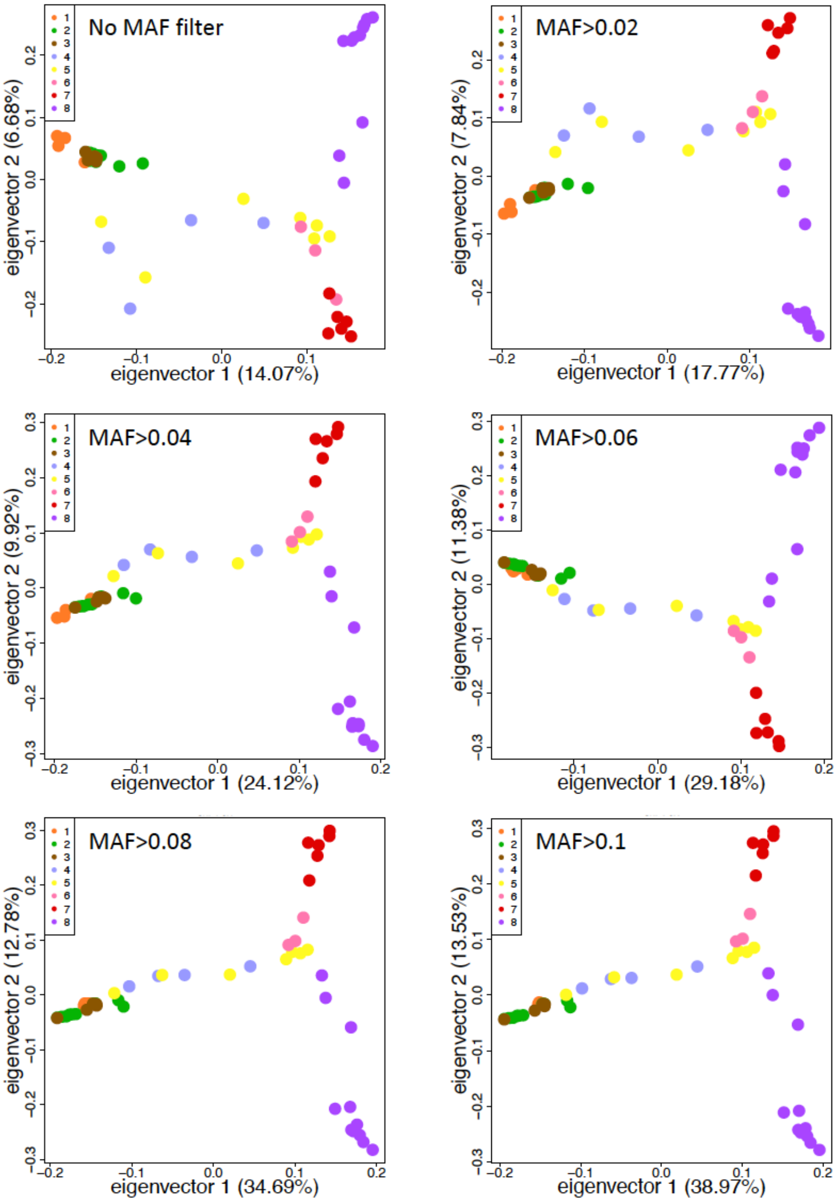

Supplemental Text Fig. 5. PCA plots of PC1 vs PC2 with varying levels of MAF filtering shows similar patterns. Color scheme used is same as used in main figures for each population cluster.

A.5 Population summary statistics

Initially we calculated population level nucleotide diversity in Stacks by reading the filtered VCF file. These values may be biased since they rely solely on variant sites. Therefore, nucleotide diversity was calculated directly from the output of gstacks using the populations command in

Stacks. Stacks does not allow for filtering SNPs by quality or read depth, however we did filter with a MAF=0.05 to match as close to our filtering parameters with VCFtools as possible. The calculated values for nucleotide diversity from radtags with no filtering, radtags with a MAF=0.05, and from the filtered VCF file are shown in **Supplemental Text Fig. 6**. Results of nucleotide diversity agree with those based on Heterozygosity estimations that SI clusters 4,5,6 in the AHZ have the highest diversity, while SC clusters 1,2,8 have the lowest.

	Gstacks no filtering		Gstacks basic filtering		VCF post filtering	
# Pop ID	Number of sites	Nucleotide diversity (π)	Number of sites	Nucleotide diversity (π)	Number of sites	Nucleotide diversity (π)
1	42,209,867	0.0023	33,048	0.00182	93,009	0.08746
2	47,794,663	0.00182	22,037	0.00159	93,127	0.0688
3	42,890,007	0.00306	11,694	0.00235	93,114	0.10759
5	47,576,974	0.00463	27,961	0.00378	93,121	0.17447
4	40,384,727	0.00403	34,692	0.00284	92,527	0.12927
6	39,070,433	0.0043	7,904	0.00303	93,024	0.13925
7	44,242,474	0.00339	31,175	0.00258	92,867	0.10839
8	46,100,220	0.00297	70,867	0.00251	93,129	0.10105
S.pennellii	40,642,690	0.00352	147,320	0.00108	70,037	0.04367

Supplemental Text Fig. 6. Comparison of nucleotide diversity using different parameters included directly from gstacks radtags with no filtering, gstacks radtags with a MAF=0.05, and the filtered VCF file.

A.6 TreeMix analysis

To investigate historical migration patterns, a phylogenetic co-variance based approach modeled in the software TreeMix v1.13 (Pickrell and Pritchard 2012) was used. Samples were separated into the eight categories identified by SNAPP plus the outgroup. Overall, zero to ten migration events were tested with a likelihood ratio test done to determine which migration events were significant **(Fig. S8B)**. Variance explained upon no migration and addition of individual migration events was obtained using the R function *get_f* in Treemix. This analysis suggested presence of seven migration events (seven vs six events: D-statistic 17.78, p-value=2.49e-05; eight vs seven events: D-statistic -3.76, p-value=1) **(Fig. S8B)**, however, 99.1% of the variance was explained by the phylogenetic tree relationships alone. Thus, migration was not found to have played an important role in *S. habrochaites* population structure.

A.7 Summary of the different input datasets used for each analysis

#	Analysis	Figure	Input data used
1	LEA	Fig. 1A,C	Set 1 SNPs
2	LEA*	Fig. S2A-C	Sets 1,2,3 SNPs
3	PCA	Fig. 1D	Set 1 SNPs
4	PCA*	Fig. S3	Sets 1,2 SNPs as described in figure

5	SNAPP	Fig. 2A	3965 conserved SNPs
6	SplitsTree4	Fig. 2B	3965 conserved SNPs
7	Targeted Structure	Fig. 3A	22 targeted RAD-tags with high coverage and breadth obtained from Set 3 SNPs
8	Heterozygosity	Fig. 4A	Variant calls from vcfTools
9	Heterozygosity-ANGSD*	Fig. S7C	All sites called from BAM files after reads were mapped to the genome
10	Fixation index (Fst)	Fig. 4B,C	All sites called from RAD-tags mapped to the genome and only variants in VCF
11	Absolute divergence (dxy)	Fig. 4B, Fig. S8A	All sites called from RAD-tags mapped to the genome
12	TreeMix	Fig. S8B	Set 2 but with populations coded based on SNAPP clusters
13	Additional analyses	Supplementary Text 1	As described in the Supplementary Text document

** Analyses performed to determine robustness of inferences*

A.8 Identification of SNPs for Targeted Sanger Sequencing

Analysis of reproductive traits identified populations of significant phenotypic interest that were not included in the original population genetic analysis. Thus, 15 accessions not included in the RAD-seq study (as well as three control accessions in the RAD-seq study) **(Table 1)** were analyzed using targeted Sanger sequencing (TSS) of 22 polymorphic loci, which were selected as follows: Since Stacks 2 versions do not provide information about the breadth and coverage of individual SNPs, we used Stacks v1.44 to obtain SNP catalogs using **Set 3 SNPs** as described above. Custom Python scripts were used to identify 36 high-confidence polymorphic loci that were present across at least 50 out of 51 accessions, had a read coverage of >10X, and were not blacklisted by the *populations* module for STRUCTURE analysis. The broad coverage across almost all accessions was intended to ensure most of them would be captured in any novel set of accessions using targeted sequencing. Twenty four of these 36 loci were randomly selected and using their genomic locations and 100 base regions on either side of the 100 bp RAD-tag were extracted for primer design. Amplicons could be successfully obtained for 22 loci.

A.9 Description of each SC group

SC-1: This group contains the low-expression *hab-7* S-RNase allele, expresses HT protein, does not possess pollen- or pistil-side interpopulation barriers*, possesses UI barriers, is illustrated as green in **Fig. 3A,B**. Accessions include LA1266, LA1264 (but see below), LA2119, LA2128, LA1252, LA2016.

SC-2: This group contains low-expression *LhgSRN-1* S-RNase allele, expresses HT protein, possesses pollen-side interpopulation barriers, does not possess pistil-side interpopulation barriers, possesses UI barriers, is illustrated as orange in **Fig. 3A,B**. Accessions include LA4656, LA1624, PI129157, LA1625, PI34417, LA0407.

SC-3: This group contains low-expression *LhgSRN-1* S-RNase allele, does not express HT protein, does not possess pollen- or pistil-side interpopulation barriers, does not possess UI barriers, is illustrated as orange in **Fig. 3A,B**. Single known accession is LA1223.

SC-4: This group contains expressed but low-activity hab-6 S-RNase allele, expresses HT protein, possesses pistil-side interpopulation barriers, three southernmost accessions LA1918, LA1927 and LA1928 possess pollen-side interpopulation barriers, all possess UI barriers, is illustrated as purple in **Fig. 3A,B**. Accessions include LA1560, LA1753, LA1691, LA1928.

SC-5: In this group, S-RNase expression is not detected, the S-RNase allele is unknown, expresses HT protein, does not either possess pollen- or pistil-side interpopulation barriers, possesses UI barriers, population structure groups with SI and MP accessions in southwest Ecuador, illustrated as blue in **Fig. 3A, B**. Accessions include LA2101, LA2860, LA2859.

SC-6: In this group, S-RNase expression is not detected, the S-RNase allele is unknown, expresses HT protein, does not possess either pollen- or pistil-side interpopulation barriers, possesses UI barriers, population structure is unique (red/green/orange) in **Fig. 3A,B**. Accessions include LA4654, LA4655.

Accessions exhibiting possible SC group hybridization in central Ecuador:

- PI309515 (SC-2/SC-3) contains the *LhgSRN-1* S-RNase allele, and its pollen tubes are rejected by SI styles like the SC-2 group, but it is like SC-3 because it does not express HT protein and does not have UI barriers.
- LA2144 (SC-1/SC-2) individuals are segregating for the *LhgSRN-1* and *hab-7* S-RNase alleles, but plants were too diseased to do additional phenotyping for Table 1
- PI251305 (SC-1/SC-2/SC-3) individuals are segregating for the *LhgSRN-1* and *hab-7* S-RNase alleles, do not express HT protein (like SC-3) but does have UI barrier, and, most surprising, this accession expresses the normally non/low-expressed hab-7 protein

- LA1266 and LA1264 exhibit all SC-1 reproductive traits but according to population structure in **Fig. 3A,B** (half blue, half green) these accessions may exhibit hybridization with the SC-5 group

  *pollen side barrier = pollen tubes of the accession are rejected by styles of SI or MP accessions, pistil-side barrier = styles of the accession reject SC-2 group pollen tubes

References

Korneliussen TS, Albrechtsen A, Nielsen R. 2014. ANGSD: Analysis of Next Generation Sequencing Data. *BMC Bioinformatics* 15:356.

Linck E, Battey CJ. 2019. Minor allele frequency thresholds strongly affect population structure inference with genomic data sets. *Mol. Ecol. Resour.* 19:639–647.

Maruki T, Lynch M. 2017. Genotype Calling from Population-Genomic Sequencing Data. *G3 Genes Genomes Genet.* 7:1393–1404.

Pickrell JK, Pritchard JK. 2012. Inference of population splits and mixtures from genome-wide allele frequency data. *PLoS Genet.* 8:e1002967.
